# A matrix of heterobimetallic complexes for interrogation of hydrogen evolution reaction electrocatalysts†
†Electronic supplementary information (ESI) available: Experimental, spectroscopic, additional electrochemical and computational details, X-ray crystallographic data (CIF) from the structure of the complexes **[Ni–Fe]^0^**, **[Ni_2_–Fe_2_]^2+^**, **[Ni_2_–Fe]^+^**, and computational coordinates are available. CCDC crystallographic data for the complexes **[Ni–Fe]^0^**, **[Ni_2_–Fe_2_]^2+^** and **[Ni_2_–Fe]^+^** were deposited in the Cambridge Crystallographic Data Centre. CCDC **[Ni–Fe]^0^** (CCDC 1045461), **[Ni_2_–Fe_2_]^2+^** (CCDC 1045460) and **[Ni_2_–Fe]^+^** (CCDC 1565539). For ESI and crystallographic data in CIF or other electronic format see DOI: 10.1039/c7sc03378h


**DOI:** 10.1039/c7sc03378h

**Published:** 2017-10-12

**Authors:** Pokhraj Ghosh, Shengda Ding, Rachel B. Chupik, Manuel Quiroz, Chung-Hung Hsieh, Nattami Bhuvanesh, Michael B. Hall, Marcetta Y. Darensbourg

**Affiliations:** a Department of Chemistry , Texas A & M University , College Station , TX 77843 , USA . Email: marcetta@chem.tamu.edu; b Department of Chemistry , Tamkang University , New Taipei City , Taiwan 25157

## Abstract

Experimental and computational studies address key questions in a structure–function analysis of bioinspired electrocatalysts for the HER. Combinations of NiN_2_S_2_ or [(NO)Fe]N_2_S_2_ as donors to (η^5^-C_5_H_5_)Fe(CO)^+^ or [Fe(NO)_2_]^+/0^ generate a series of four bimetallics, gradually “softened” by increasing nitrosylation, from 0 to 3, by the non-innocent NO ligands. The nitrosylated NiFe complexes are isolated and structurally characterized in two redox levels, demonstrating required features of electrocatalysis. Computational modeling of experimental structures and likely transient intermediates that connect the electrochemical events find roles for electron delocalization by NO, as well as Fe–S bond dissociation that produce a terminal thiolate as pendant base well positioned to facilitate proton uptake and transfer. Dihydrogen formation is *via* proton/hydride coupling by internal S–H^+^···^–^H–Fe units of the “harder” bimetallic arrangements with more localized electron density, while softer units convert H^–^···H^–^*via* reductive elimination from two Fe–H deriving from the highly delocalized, doubly reduced [Fe_2_(NO)_3_]^–^ derivative. Computational studies also account for the inactivity of a Ni_2_Fe complex resulting from entanglement of added H^+^ in a pinched –S^*δ*–^···H^+^···^*δ*–^S– arrangement.

## Abstract 2

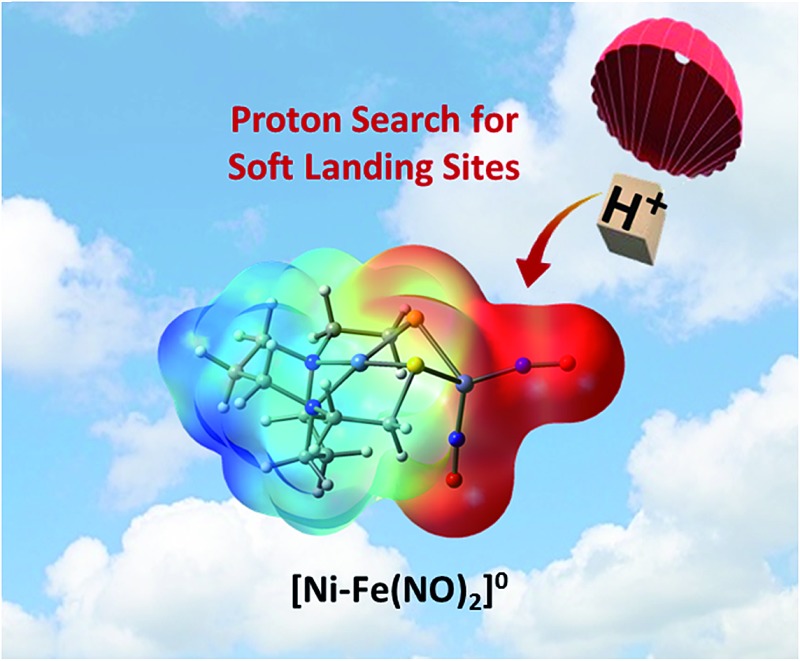
Nitrosyls as electron reservoirs guide protons to favorable sites in bimetallic HER catalysts.

## Introduction

From protein crystallography the bimetallic active site structures in enzymes such as [NiFe]-, [FeFe]-hydrogenases (H_2_ase), CO-dehydrogenases and acetyl coA synthase (ACS) have been convincingly interpreted in terms of characteristics needed for their organometallic-like functions.^1^,^2^ Whereas most major homogeneous catalytic applications involving redox processes use precious metals that can operate as single site catalysts, the intricate molecular arrangements in nature's biocatalysts harness combinations of at least two first row transition metals, connected by sulfides or thiolates, along with Lewis acid/base sites.^3^–^5^ Over the past two decades a rich area in synthetic chemistry inspired by such natural products has developed, yielding biomimetics for insight into enzyme mechanisms. In addition the link between the [NiFe]- and [FeFe]-H_2_ase active sites and base metal, sustainable catalysts for the Hydrogen Evolution Reaction (HER) holds promise for the production of H_2_ from “solar” (photovoltaic) electrons *via* electrocatalysis.^6^ Specific efforts have been directed towards the use of metallodithiolates from MN_2_S_2_ complexes as bidentate donor ligands (readily deduced from the structure of the ACS enzyme active site), that bind to receiver metal units *via* bridging dithiolates.^7^–^25^ The electronic requirements of the thiolate sulfurs have a steric consequence in the butterfly M(μ-SR)_2_M′ cores that are seen in the H_2_ase active sites, placing M and M′ within close proximity.^26^

**Fig. 1:**
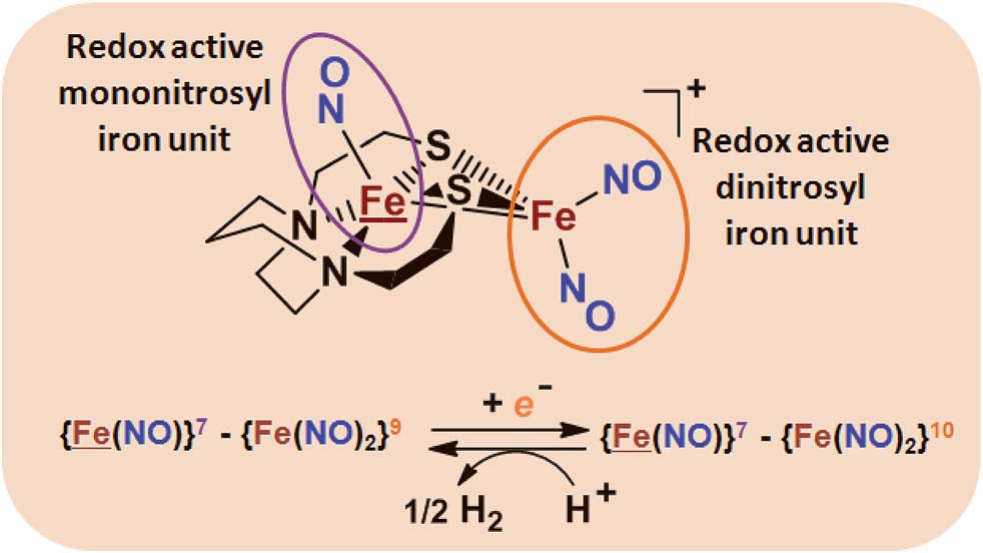
Structure and redox activity of 
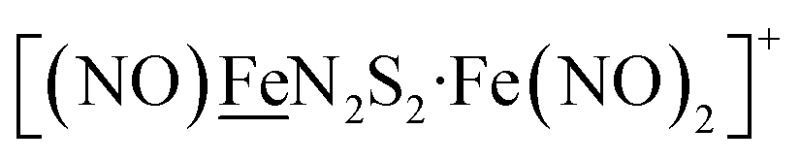
, 
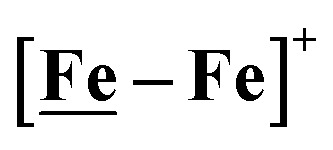
; protonation of the one-electron reduced diiron complex yields H_2_.^8^

The advancement of chemistry *via* structure/function analysis of sets of compounds with well-known differences in composition and structure is a challenge in the complicated area of HER electrocatalysis. Nonetheless the metallodithiolate-as-synthon approach, inspired from the ACS active site, permits modular design that includes some features of the bimetallic [NiFe]- and [FeFe]-H_2_ase active sites beyond the obvious dithiolate core structures. An initial foray explored the properties of the diiron, trinitrosyl complex shown in Fig. 1.^8^,^27^ With it we intended to exploit the redox-activity of 
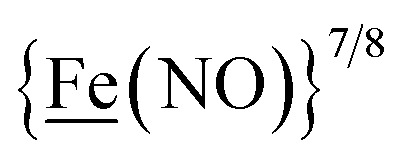
 in the 
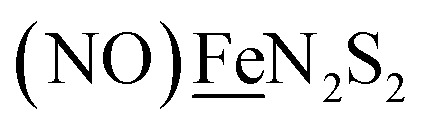
 metalloligand bound to a redox-active {Fe(NO)_2_}^9/10^, iron dinitrosyl unit. Electrochemical studies of 
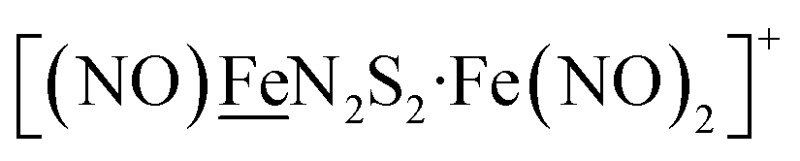
, 
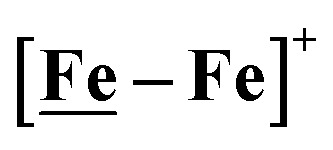
, Fig. 1, found two single-electron, reversible reduction events, –0.78 V and –1.33 V, assigned to {Fe(NO)_2_}^9/10^ and 
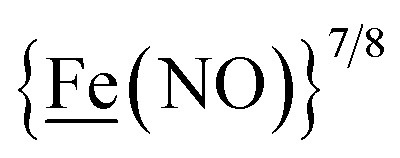
 couples, respectively.^8^ The Fe of the 
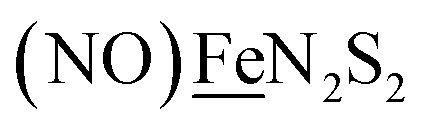
 metalloligand is herein distinguished as 
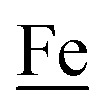
; the electron count of the iron nitrosyl units uses the Enemark–Feltham approach.^28^ Consistent with the stoichiometric reaction shown in Fig. 1, the {Fe(NO)_2_}^9/10^ couple, at –0.78 V, was the catalytically active center for electrochemical proton reduction in the presence of strong acid, HBF_4_·Et_2_O. Although modest in overpotential and TOF, electrocatalysis for H_2_ production was observed at this potential; preliminary computational studies indicated that a hydride-bound {Fe(NO)_2_}^8^ could likely be a transient intermediate, however the overall H_2_ releasing mechanism was at that stage incomplete.^8^ Interestingly, the second reduction process, related to the more negative 
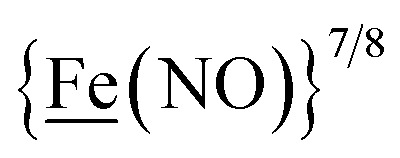
 couple, showed a current response to added weak acid, however H_2_ was not detected. Computational study attributed this to a non-productive reduction event with protonation on the nitrosyl, which terminates the catalytic cycle.^8^

**Fig. 2:**
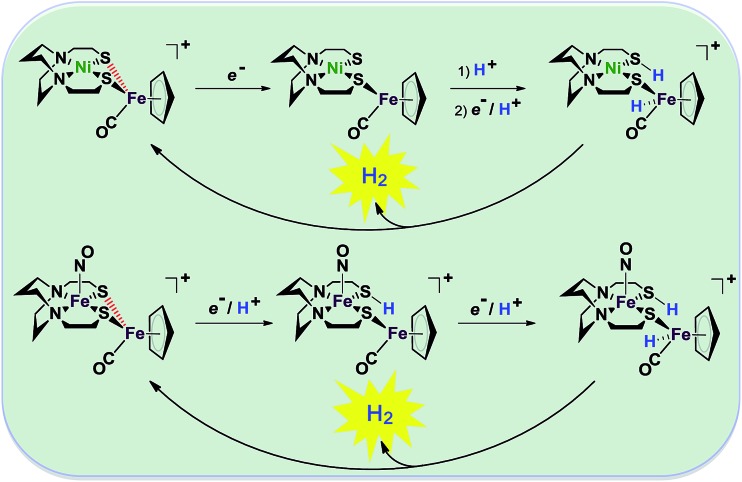
Abbreviated computational mechanisms for electrocatalysis of H_2_ production by the **[Fe–Fe′]^+^** and **[Ni–Fe′]^+^** electrocatalysts.^9^ Shown in red is the Fe–S bond that undergoes reductive bond cleavage.

We have made analogues of the diirion trinitrosyl complex making use of NiN_2_S_2_ and 
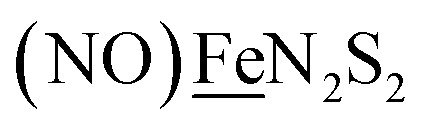
 metallodithiolates in combination with η^5^-C_5_R_5_ derivatives (R = H, CH_3_),^9^,^10^ of Fe^II^ shown in Fig. 2. The large differences in reduction potential of the MN_2_S_2_ ligands, with the d^8^-Ni^II^ being more negative because of a more rigid, less polarizable electronic structure as compared to the delocalized 
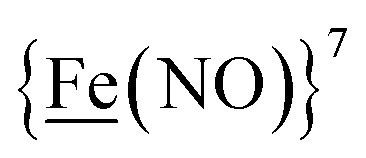
 unit, of greater electronic flexibility, inspired the labels of “hard” for the former MN_2_S_2_ unit, and “soft” S-donor unit for the latter. The Fe-receivers also differ in electronic flexibility and their ease of electron uptake, the term “soft” describing the highly delocalized {Fe(NO)_2_}^9^ unit, and the indefinite oxidation state of the iron, as compared to the definite Fe^II^ of the η^5^-C_5_R_5_, “hard” receiver derivatives.^8^,^9^ The hard receiver unit, (η^5^-C_5_H_5_)Fe(CO)^+^, is herein distinguished from the soft Fe(NO)_2_ unit by Fe′ and Fe, respectively.

Notable from the computational approach that guided the interpretation of electrochemical events of the **[Ni–Fe′]^+^** and 
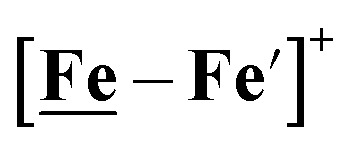
 complexes in the presence of acid was the indication of a reductive iron–sulfur bond cleavage (shown in red, Fig. 2) that converted the bidentate dithiolate into a monodentate S-donor, thus creating a transient frustrated Lewis pair, *i.e.*, yielding reactive sites for proton and electron uptake on the free thiolate and the open site on iron, respectively, see Fig. 2.^9^ In this way, complexes, that do not have an amine pendant base for proton uptake and storage, or open sites on iron for a hydride, as in the [FeFe]-H_2_ase active site,^29^–^33^ adjust their structures to accommodate coupled electron/proton uptake. While the mild potential for the first EC process for the 
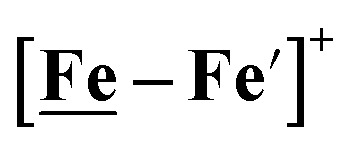
 complex required both proton/electron uptake for genesis of the pendant base, the more negative potential that reduces the **[Ni–Fe′]^+^** labilizes the sulfur and creates an Fe^III^–H at the first reduction, Fig. 2.^8^,^9^

In this report we provide a more complete matrix of MN_2_S_2_–Fe complexes of electrocatalytic potential for experimental and computational analysis. Specifically a redox innocent (“hard”) metalloligand, NiN_2_S_2_, of more negative reduction potential, is incorporated in place of “soft” 
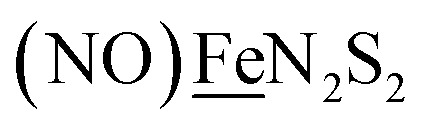
. The thus generated [Ni^II^N_2_S_2_·Fe(NO)_2_]^0/+^, a “hard”/“soft” complex may be compared to the other members of the matrix. The solid state structures of [Ni^II^N_2_S_2_·Fe(NO)_2_]^0/+^ in two redox levels and characteristics as an electrocatalyst (robustness and turnover frequency), for proton reduction are also described. Computational study, addresses the diversity of geometries of di- and poly-metallic compounds containing N_2_S_2_ metalloligands by inspecting the versatile bonding orbitals of the metalloligands. The computational mechanisms contrast the working electrocatalysts against a non-working analogue by exploring possible intermediates in the proposed catalytic cycles. Here important roles for hemi-labile and redox active ligands are revealed.

## Results and discussion

### Synthesis and characterization

**Scheme 1:**
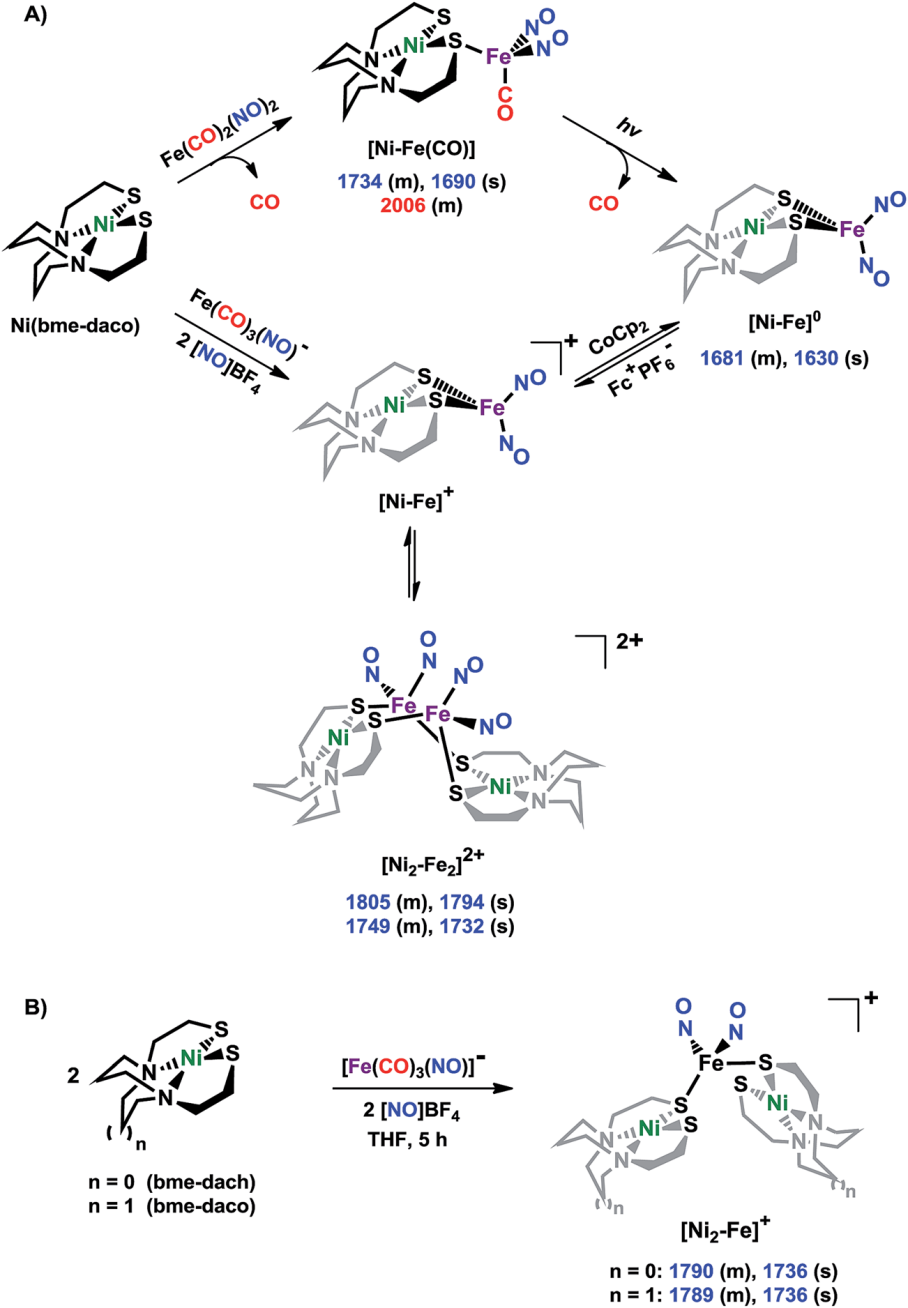
(A) The syntheses of **[Ni–Fe]^0^** and **[NiFe(CO)]^0^** as neutral complexes, and **[Ni_2_–Fe_2_]^2+^** and (B) **[Ni_2_–Fe]^+^** as BF_4_^–^ salt. The IR values (in cm^–1^) of CO and NO are in red and blue, respectively.

Shown in Scheme 1A are the synthetic routes to NiN_2_S_2_·Fe(NO)_2_, **[Ni–Fe]^0^**, and its one-electron oxidized analogue, **[Ni–Fe]^+^**, isolated and crystallographically characterized as a dimer, [NiN_2_S_2_·Fe(NO)_2_]_2_^2+^ or **[Ni_2_–Fe_2_]^2+^**, (N_2_S_2_ = *N*,*N*-bis(2-mercaptoethyl)-1,5-diazacyclooctane or bme-daco). Infrared values listed for the diatomic ligands were recorded in CH_2_Cl_2_ or THF solution. Freshly prepared Fe(CO)_2_(NO)_2_ in THF readily reacts with NiN_2_S_2_ at 22 °C, with replacement of one CO, releasing the second CO under photolysis, or within 20 min at 40 °C, thus converting the NiN_2_S_2_ from mono- to bidentate ligand.^34^–^37^

From this approach the **[Ni–Fe]^0^** complex was isolated as a brown solid that is stable at ambient temperature under Ar. Oxidation of **[Ni–Fe]^0^** by Fc^+^PF_6_^–^ at 0 °C resulted in a color change of the THF solution from brown to dark purple with concomitant shifts of the *ν*(NO) values by an average of *ca.* 110 cm^–1^ to higher wave numbers. The reversibility of this oxidation was confirmed by reaction with cobaltocene and return to the reduced **[Ni–Fe]^0^**. The *ν*(NO) bands listed under **[Ni_2_–Fe_2_]^2+^**, Scheme 1A, reflect the presence of overlapping components which were resolved into two sets of absorbances, interpreted as a likely mixture of monomeric cation and dicationic dimer, with the set at lower values slightly less in intensity (Fig. S24†). As other experimental data, *vide infra*, as well as computational studies, indicate the predominance of monomeric **[Ni–Fe]^+^**, we postulate that the set of absorbances at slightly lower wavenumbers (as shoulders on the major bands) are due to the dimeric **[Ni_2_–Fe_2_]^2+^**. We note that the electron-spray ionization mass spectrum of **[Ni–Fe]^+^** has a parent ion with isotopic bundle distribution at *m*/*z* that is consistent with a monomeric **[Ni–Fe]^+^** rather than a dimeric **[Ni_2_–Fe_2_]^2+^**, Fig. S28.† The difference between two consecutive isotopic mass units is ∼1, rather than 0.5, which indicates the predominance of the monomer, **[Ni–Fe]^+^**, in the polar solvents in which they are soluble.

The magnetic moments of **[Ni–Fe]^+^** and **[Ni_2_–Fe]^+^** are 1.54 B.M. and 1.76 B.M., respectively, measured by Evans' method at room temperature in CD_2_Cl_2_. This is consistent with the presence of a single unpaired electron, Fig. S1 and S2.† The EPR spectra for both complexes display the isotropic *g* = 2.03 signal that is prototypical of the {Fe(NO)_2_}^9^ unit, Fig. S21 and S22,† respectively. The 77 K EPR spectrum of the **[Ni–Fe]^+^** displayed fine structure requiring two signals for simulation: A major isotropic signal of *g* = 2.035 showed coupling with nitrogen of *A*(^14^N) = 32.74 MHz and a minor anisotropic signal had parameters of *g*_*xyz*_ = 2.183, 2.012, 1.908 and no observable hyperfine coupling, Fig. S21.†

X-ray diffraction quality crystals of the oxidized NiFe compound were obtained from the one-pot reaction of equimolar NiN_2_S_2_ and (putative) [Fe(CO)_2_(NO)_2_]^+^ (prepared *in situ* by reacting [Fe(CO)_3_(NO)]^–^ with two equivalents of [NO]BF_4_)^38^ in CH_2_Cl_2_ at ambient temperature, Scheme 1. A third Ni–Fe complex, **[Ni_2_–Fe]^+^**, was obtained on combining NiN_2_S_2_ with [Fe(CO)_2_(NO)_2_]^+^ in 2 : 1 ratio in THF solution, Scheme 1B. X-ray quality crystals of this trimetallic as its BF_4_^–^ salt were obtained from hexane/THF layering at –28 °C. Its *v*(NO) IR spectral features are typical of monomeric DNICs in the {Fe(NO)_2_}^9^ redox level.

### X-ray diffraction studies

**Fig. 3:**
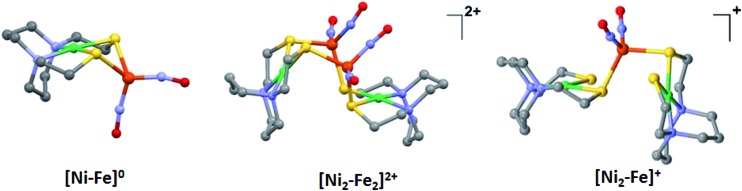
Molecular structures of (a) **[Ni–Fe]^0^**, (b) **[Ni_2_–Fe_2_]^2+^** and (c) **[Ni_2_–Fe]^+^** from X-ray diffraction. The BF_4_^–^ counter ions of **[Ni_2_–Fe_2_]^2+^** and **[Ni_2_–Fe]^+^** are omitted for clarity.

The molecular structures of the heterometallic complexes **[Ni–Fe]^0^**, **[Ni_2_–Fe_2_]^2+^** and **[Ni_2_–Fe]^+^** are shown in Fig. 3. The bimetallic complex **[Ni–Fe]^0^**, exhibits an overall butterfly-like Ni(μ-SR)_2_Fe core, analogous to the report of Pohl *et al.*, where an open chain N_2_S_2_ ligand chelated the Ni^II^.^36^ The converging lone pairs (see below) on the *cis*-dithiolates engage in bidentate binding and impose a hinge angle (the intersection of the best N_2_S_2_ plane with the S_2_Fe plane) of *ca.* 117°, *vis-à-vis* constricting the ∠S–Ni–S angle by *ca.* 4° compared to the free metalloligand.^39^ The two nitrosyl units bound to the pseudo tetrahedral iron center are slightly bent towards each other, in an “attracto” orientation;^40^ the average ∠Fe–N–O angle is ∼163.8°. The Ni···Fe distance of 3.001 (2) Å, is slightly longer (by 0.022 Å) than that obtained in the Pohl, *et al.* structure.^36^

The oxidized NiFe complex crystallizes as dimeric **[Ni_2_–Fe_2_]^2+^** with two BF_4_^–^ anions; two dinitrosyl iron units are bridged by two NiN_2_S_2_ metalloligands. The tetrahedral geometry about each Fe(NO)_2_ unit is thus completed by two thiolates from different NiN_2_S_2_ metalloligands, thus generating an abbreviated paddlewheel, or propeller type, complex seen in examples of nickel–gold tetrametallic complexes.^41^ Likewise, the molecular structure of **[Ni_2_–Fe]^+^** demonstrates that one Fe(NO)_2_ unit bridges two NiN_2_S_2_ metalloligands, each acting as a monodentate ligand. As shown in the **[Ni_2_–Fe]^+^** structure, Fig. 3, the free unbound thiolates of two NiN_2_S_2_ units are transoid to each other. The addition of a second Fe(NO)_2_^+^ unit to generate the dication, **[Ni_2_–Fe_2_]^2+^**, would require rotation about one Fe–S bond in order to align the two metalloligands.

The average Ni···Fe distances in **[Ni_2_–Fe_2_]^2+^** and **[Ni_2_–Fe]^+^**, are 3.680 (2) Å and 3.521 (2) Å, respectively, and are longer than in the **[Ni–Fe]^0^** reduced complex by *ca.* 0.5 Å. The Ni^II^ maintains a square planar geometry in the reduced and oxidized complexes with a displacement of no more than 0.1 Å from the N_2_S_2_ best plane. Overall these structures demonstrate the impressive adaptability of the NiN_2_S_2_ metalloligands, and their potential to template clusters through S-based aggregation.^7^

### Computational structural study

This computational section uses density functional theory (DFT) analysis to address the structural question in particular that was raised by the X-ray diffraction report: is there an electronic factor that governs the different μ_2_-SR binding modes found in the three forms of NiFe heterometallic aggregates? The functional/basis set combination, TPSS/6-311++G(d,p), and natural bond orbital (NBO) analysis were applied to the computational structural modeling of the free metalloligand NiN_2_S_2_ and its derivatives **[Ni–Fe]^0^**, **[Ni_2_–Fe_2_]^2+^**; more details of the computational methodology is available in ESI.†

#### The divergent or convergent orientation of S lone pairs of NiN_2_S_2_ metalloligand and influences on structures of NiN_2_S_2_·M′ heterobimetallics

**Fig. 4:**
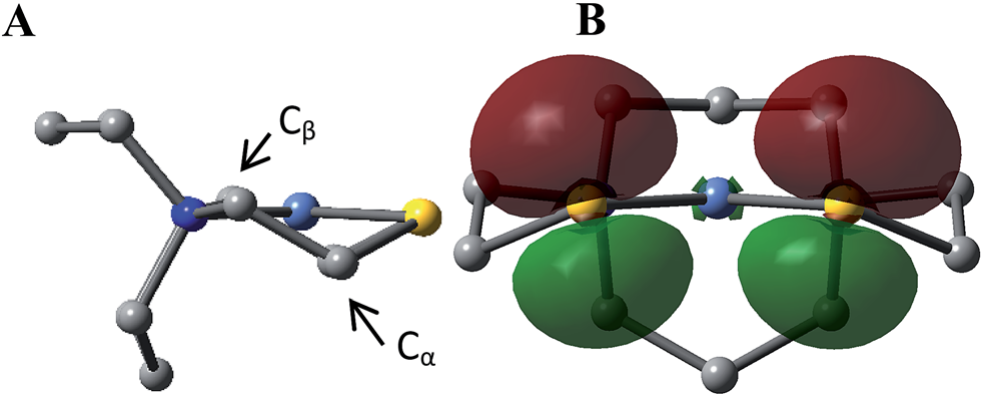
(A) The geometry of a free metalloligand NiN_2_S_2_ and (B) its two 3p lone pairs presented one on each sulfur (contour plots at isovalue = 0.05 a.u. by NBO analysis). Note the relative positions of the S–C_α_/S–Ni bonds and the 3p-lone pair.

Traditional bidentate ligands such as diphosphines, diamines and bipyridyls have a single lone pair on each donor site. These lone pairs are positioned on orbitals originating from sp^*x*^-hybridization and are highly directional.^42^ They provide fixed binding orientations that correspond one-to-one with the coordination sites of the metal. In contrast, the binding between the sulfurs of the metallothiolate NiN_2_S_2_ and an exogeneous metal are more geometrically flexible because of the multiple S lone pairs. From NBO bonding analysis, sulfur in the NiN_2_S_2_ metalloligand is found to use mainly p orbitals for bonding to Ni and C.^43^,^44^ For example, in a free NiN_2_S_2_, p character makes up 83% and 86% of the S contributions in the S–Ni bonds and S–C_α_ bonds (C_α_ and C_β_ refer to the C_2_H_4_ linker connecting S and N where C_α_ is directly bound to S, Fig. 4A), which leaves one lone pair in a p orbital and another in an s-dominated orbital on each S. Because a receiver group, a Fe(NO)_2_ unit in our case, may bind to either lobe of the p lone pair(s), whose orientation is determined by the Ni–S–C_α_ torsion angle, a diversity of structures results.^7^,^26^

The orientation of this remaining p lone pair in the NiN_2_S_2_ metalloligand is determined by the NiN_2_S_2_ metalloligand's Ni–S–C_α_–C_β_–N five-membered rings that adopt a non-planar envelope conformation like cyclopentane. The C_α_ carbon, the “flap” of the envelope conformation, puckers towards one side or the other of the N_2_S_2_ plane, Fig. 3. Fig. 4 shows how this puckering tilts the remaining 3p-lone pair on each sulfur from the normal to the N_2_S_2_ plane. This tilt causes two p-orbital lobes (green lobes) to converge on the side to which the flap puckers, while the red lobes diverge on the opposite side. The orientation of the added Fe(NO)_2_ receiver unit(s), will be thus determined by such directional property of the donor p lone pairs. The structure of the reduced monomer **[Ni–Fe]^0^** shows the Fe(NO)_2_ fragment is on the same side as the flap; while in the oxidized dimer **[Ni_2_–Fe_2_]^2+^** the flap and the Fe(NO)_2_ fragment(s) appear on different sides of each N_2_S_2_ plane, thus, binding to the other end of the p lone pair. Based on the analysis above, the converging lobes of the p donor lone pairs maximize contact to the Fe(NO)_2_ unit in the monomer **[Ni–Fe]^0^**, while the diverging lobes are preferred by two bridging Fe(NO)_2_ units between two metalloligands in the dimer **[Ni_2_–Fe_2_]^2+^**. The utilization of the divergent lobes apparently lessens the steric repulsion between Fe(NO)_2_ units. In summary, the binding position of the Fe(NO)_2_ unit with respect to the flap in the Ni–S–C_α_–C_β_–N five-membered rings are correlated by the competition between chemical bond directionality of the binding sulfurs and steric repulsion of the receiver units.

### Electrochemistry

The cyclic voltammograms of **[Ni–Fe]^+^** (in CH_2_Cl_2_), **[Ni_2_–Fe]^+^** (in CH_3_CN), as BF_4_^–^ salts, and **[Ni–Fe]^0^** (in CH_2_Cl_2_), were recorded under Ar at 22 °C, and referenced to Fc^0/+^ (*E*_1/2_ = 0.0 V) as an internal standard. Both the neutral complex **[Ni–Fe]^0^** and the cationic analogue, **[Ni–Fe]^+^**, used in the CV studies as its BF_4_^–^ salt, displayed reversible reduction events at *ca.* –0.73 V (CH_2_Cl_2_), assigned to the {Fe(NO)_2_}^9/10^ couple; see Fig. S13–S15† for CV scans. The NiFe complexes also present two irreversible oxidation events at *ca.* –0.10 V and *ca.* +0.45 V, with minor differences in intensities according to the neutral or cationic sources. Both of these events are assumed to be S-based.

In CH_3_CN, the trimetallic complex **[Ni_2_–Fe]^+^**, showed a reversible event at, *E*_1/2_ = –0.75 V, assigned to the {Fe(NO)_2_}^9/10^ couple and an irreversible oxidation event at *E*_1/2_ = –0.05 V, see Fig. S16.† The *E*_1/2_ value for the {Fe(NO)_2_}^9/10^ couple, is anodically shifted by *ca.* 30 mV relative to the 1 : 1, NiFe complexes, resulting from the greater electron donation of two NiN_2_S_2_ centers to the Fe(NO)_2_ redox marker. The scan rate dependences of the {Fe(NO)_2_}^9/10^ couple for all three complexes support the assignments to reversible or quasi-reversible as described above, see Fig. S17–S19.†

#### Cyclic voltammetry and response to added acid

**Fig. 5:**
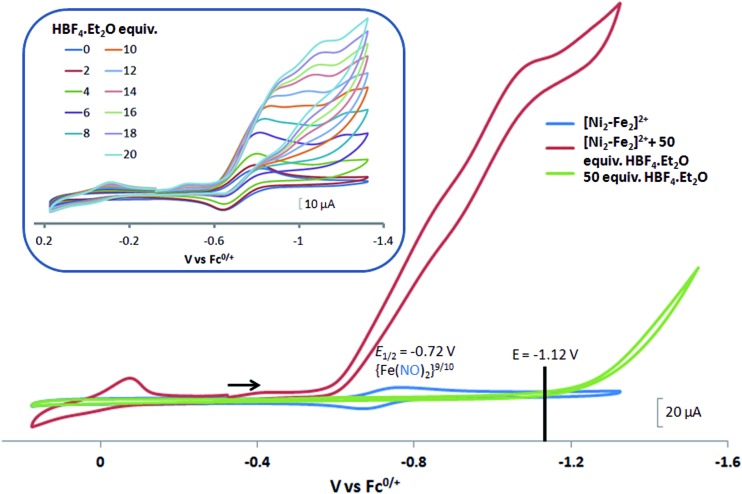
Cyclic voltammograms of 2.0 mM CH_2_Cl_2_ solutions of **[Ni_2_–Fe_2_]^2+^** (or **[Ni–Fe]^+^**(blue)); with 50 equiv. (0.1 M) of added HBF_4_·Et_2_O (red); and, for reference, 50 equiv. (0.1 M) of HBF_4_·Et_2_O (green). The black line indicates the constant potential applied for bulk electrolysis, –1.12 V. Inset: cyclic voltammograms of **[Ni_2_–Fe_2_]^2+^** (or **[Ni–Fe]^+^**) with 2 to 20 equiv. aliquots of HBF_4_·Et_2_O. Crystalline **[Ni_2_–Fe_2_]^2+^** was dissolved as its BF_4_^–^ salt, in 0.1 M ^*t*^Bu_4_NPF_6_ as supporting electrolyte, with a glassy carbon electrode at scan rate of 200 mV s^–1^. Note: equivalents of HBF_4_·Et_2_O was calculated with respect to the dimeric **[Ni_2_–Fe_2_]^2+^**.

Electrochemical studies of **[Ni–Fe]^+^** and **[Ni_2_–Fe]^+^**were carried out in presence of HBF_4_·Et_2_O under a N_2_/Ar atmosphere. For comparison the 
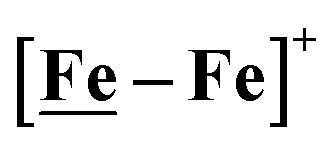
 complex was examined under similar experimental conditions. Sequential addition of HBF_4_·Et_2_O to a CH_2_Cl_2_ solution of **[Ni–Fe]^+^** (2 mM **[Ni_2_–Fe_2_]^2+^**) showed an increase in the cathodic current at the {Fe(NO)_2_}^9/10^ redox event at –0.73 V. The initial cathodic current response at –0.8 V saturates with ∼20 equivalents of the acid, Fig. 5 (inset). A second rise in cathodic current at –1.10 V, commences upon addition of >12 equivalents of the acid, which continues to rise as the catalytic current response, Fig. 5. The first response is attributed to the reduction of **[Ni–Fe]^+^** followed by a protonation. The second response is assigned to the up-take of another electron by the reduced and protonated counterpart of **[Ni–Fe]^+^**. The mechanism below connects the successive protonation to the production of H_2_, thus closing the catalytic cycle. Overlays of this response of the NiFe complex in presence of 50 equivalents of HBF_4_·Et_2_O (0.1 M), as well as the CV of the free acid, are shown in Fig. 5. The catalytic H_2_ produced was confirmed by applying a constant potential at –1.12 V for 60 min (black bold line in Fig. 5), and analysis of the headspace by gas chromatography. The H_2_ was quantified by an average of two consistent constant potential coulometry experiments with subtraction of the H_2_ produced from the free acid.^9^,^10^ The nitrosylated compounds **[Ni–Fe]^+^** and 
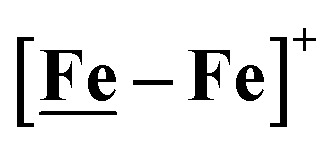
 were found to have low turnover numbers and faradaic efficiencies, 68 ± 2% and 58 ± 1%, respectively, for H_2_ production. In contrast the **[Ni–Fe′]^+^** gave a faradeic efficiency of *ca.* 96%. We assume that the former involves alternate protonation pathways, particularly at NO, that lead to degradation and hence low F.E. In addition the TON for the robust **Ni–Fe′** complex in 50 equiv. of TFA, measured at –1.73 V and over a period of 8 h was found to be 6.7, assuring catalytic proton reduction. The electrocatalytic response of the reduced complex, **[Ni–Fe]^0^** in the presence of HBF_4_·Et_2_O, is, as expected, the same as **[Ni–Fe]^+^** and is shown in Fig. S20-B.†

Following the approach of Helm and Appel,^45^ and Wiese,^46^ the turnover frequency (TOF) as calculated from the CV experiment for **[Ni–Fe]^+^**, was 39.7 s^–1^, which is slightly better than the 
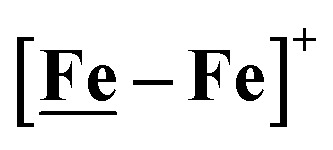
 complex, 26.7 s^–1^, calculated under similar experimental conditions. The **[Ni–Fe]^+^** shows a saturation of the more negative catalytic current upon addition of 80 equivalents of HBF_4_·Et_2_O, *i.e.*, ∼0.16 M CH_2_Cl_2_ solution. Notably, the behavior of the 
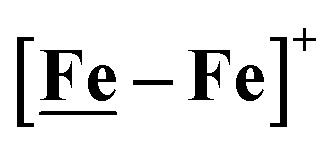
 complex is similar, and further addition of acid leads to decomposition of both catalysts. The precise calculation of overpotential is indeterminable as the thermodynamic potential (*E*_HBF_4_/H_2_,BF_4_^–^_) of 0.1 M HBF_4_·Et_2_O in CH_2_Cl_2_ is unavailable.^47^ Using the thermodynamic potential of HBF_4_·Et_2_O in acetonitrile (–0.26 V),^48^,^49^ an estimate of the overpotential of **[Ni–Fe]^+^** and 
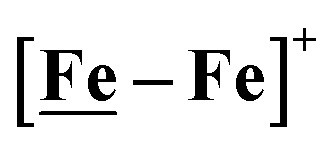
 were 711 mV and 660 mV, respectively, which are lower than those of the **[Ni–Fe′]^+^** and 
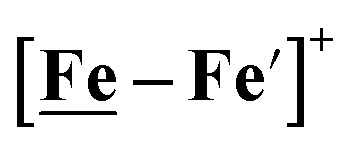
 electrocatalysts by over 220 mV.

**Fig. 6:**
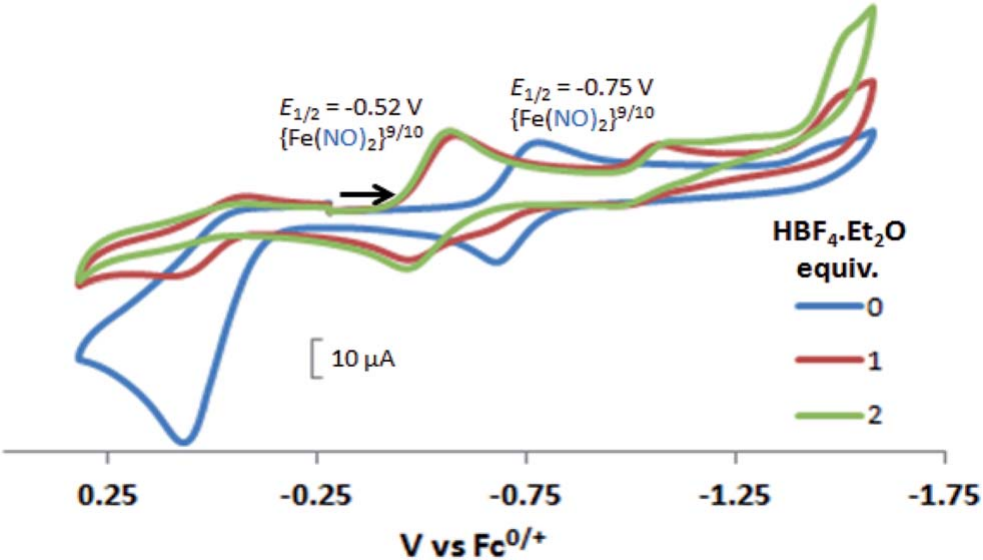
Cyclic voltammograms of 2.0 mM CH_3_CN solution of **[Ni_2_–Fe]^+^** (blue), with 1 and 2 equiv. of added HBF_4_·Et_2_O (red and green, respectively). Note: the N_2_S_2_ ligand used in this compound is bme-dach.

In contrast to the NiFe complexes, addition of HBF_4_·Et_2_O to a 2.0 mM CH_3_CN solution of **[Ni_2_–Fe]^+^**, (the N_2_S_2_ ligand used in this electrochemical study is bme-dach) did not show an increase in the cathodic current at –0.75 V, the reversible {Fe(NO)_2_}^9/10^ redox event. Instead, a new reversible redox event at *E*_1/2_ = –0.52 V, appeared upon addition of two equivalents of HBF_4_·Et_2_O with a concomitant disappearance of the original redox process, Fig. 6. Further addition of acid resulted in electrode fouling, Fig. S20-A.† A possible explanation, from computational chemistry, *vide infra*, for the positive 230 mV shift is that **[Ni_2_–Fe]^+^** can be protonated on its exposed thiolate sulfur by HBF_4_·Et_2_O, *vide infra*. Such would account for the greater ease of reduction for the {Fe(NO)_2_}^9/10^ couple, compared to the **[Ni_2_–Fe]^+^** complex. Supporting this conclusion is that addition of 1 equivalent of HBF_4_·Et_2_O to a CH_3_CN solution of **[Ni_2_–Fe]^+^** produced a small but definite shift of the *ν*(NO) in the IR spectrum from 1787 and 1734 cm^–1^ to 1793 and 1737 cm^–1^, Fig. S27.† In addition, the irreversible oxidation event at 0.07 V, which is assumed to be sulfur-based oxidation, shows a decrease in the anodic current upon addition of acid, indicating disulfide formation is inhibited under acidic conditions.

### Computational mechanistic study

The electrochemical study points to additional questions for computational study: (A) how do the calculated electrocatalytic mechanisms compare for the hard–soft *vs.* soft–soft donor/receiver adducts? (B) Can computational analysis clarify those cases of non-catalytic electrochemical responses to added protons? Modeling is extended to **[Ni_2_–Fe]^+^**, along with **[Ni–Fe]^0^**, **[Ni–Fe]^+^**, in various oxidation states and with multiple added protons to answer these questions.

#### Mechanistic perspectives of the proton reduction electrocatalysis by **[Ni–Fe]^+^**/**[Ni–Fe]^0^** and comparison to 
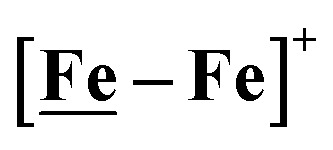


**Fig. 7:**
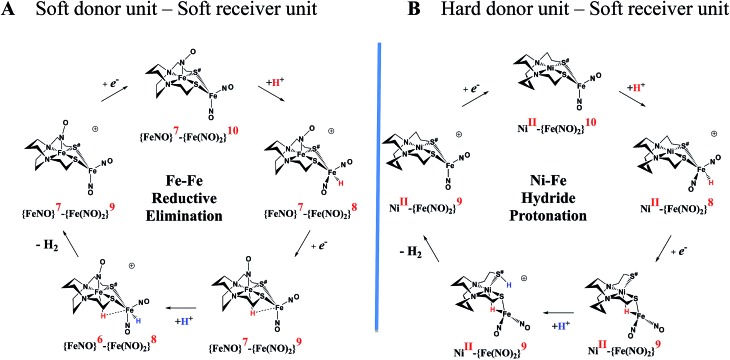
Comparative catalytic cycles for H_2_ production catalyzed by 
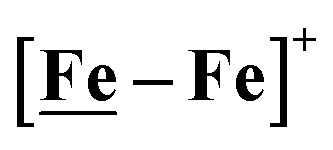
 and **[Ni–Fe]^+^**. All p*K*_a_, thermodynamic, and metric data for the two mechanisms are available in a separate report.^50^

An earlier computational analysis of a HER electrocatalysis mechanism proposed for the 
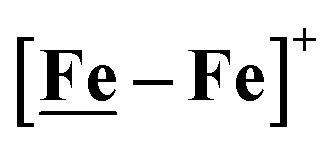
 complex, Fig. 1, focused on the first reduction event with a strong acid proton source.^8^ The successive reduction event ultimately allowed for double proton addition to the Fe(NO)_2_ unit and formation of a dihydride, panel A of Fig. 7.^8^,^50^ Making use of electron transfer from the reduced {Fe(NO)}^8^, reductive elimination from the {Fe(NO)}^6^–{Fe(NO)_2_}^8^ morphed into an η^2^-H_2_–Fe(NO)_2_, restoring {Fe(NO)}^7^–{Fe(NO)}^9^, with H_2_ formation and loss. Note that no hemi-lability of the metallodithiolate ligand^9^ is necessary here as the mechanism does not entail hydride/proton coupling to H_2_, but rather reductive elimination from two hydrides.^7^

The **[Ni–Fe]^+^** and its reduced counterpart **[Ni–Fe]^0^** are determined to be electrocatalysts at –0.73 V for H_2_ production with HBF_4_·Et_2_O, Fig. 5. [Note: The computational study finds that the **[Ni_2_–Fe_2_]^2+^**, whose dimeric structure was established in the solid state by crystallography, finds greater stability in solution as the monomeric form, **[Ni–Fe]^+^**. Experimental evidences including ESI-MS and determination of *μ*_eff_ support this thesis, *vide supra*.] The catalytic cycle is thus initiated with the monomer **[Ni–Fe]^+^** (Fig. 7B). As indicated in panel B of Fig. 7, the {Fe(NO)_2_}^9^ in the **[Ni–Fe]^+^** unit accepts the first incoming electron, followed by the first proton, to create a hydride on the now {Fe(NO)_2_}^8^ unit. Addition of a second electron activates the hemi-lability of the bridging thiolate to break one S–Fe bond, while the terminal hydride becomes bridging between Fe and Ni. The cleavage of the S–Fe dative bond essentially releases one p lone pair of the thiolate so that S can act as a pendant base to accept the second proton and guide it to a coupling position with the hydride and produce H_2_. Details of the full catalytic cycle with energetics and analysis of electronic structure evolution for both 
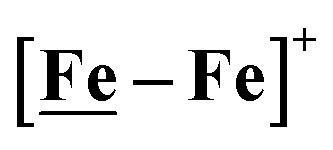
 and **[Ni–Fe]^+^** are presented in a separate report.^50^

#### Explanation for the absence of catalytic activity of **[Ni_2_–Fe]^+^**

**Fig. 8:**
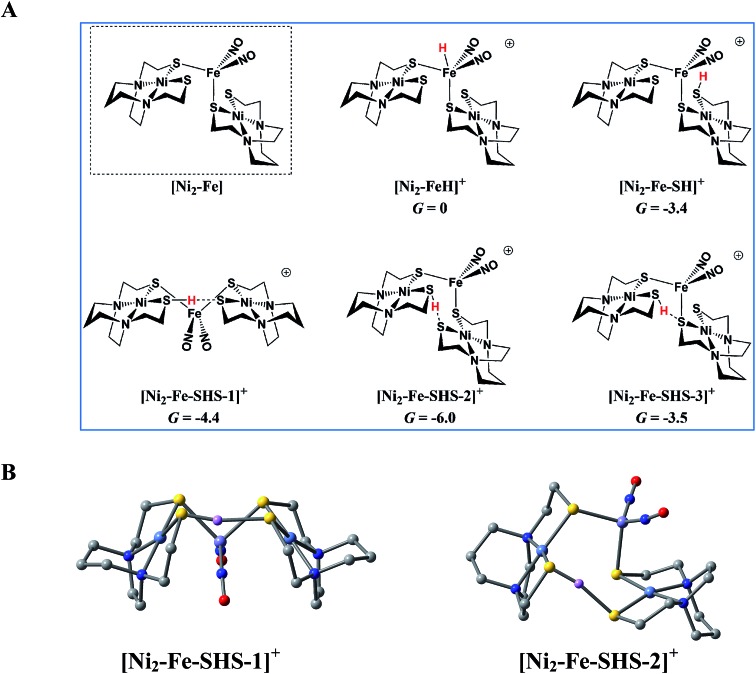
The protonation of **[Ni_2_–Fe]**, the reduced form of **[Ni_2_–Fe]^+^** and (a) possible protonated products with (b) 3D geometric presentations of selected species featuring the pinched proton. The computationally derived structures are rendered so as to show the NiN_2_S_2_ metalloligand without altering the rigidity of the N_2_S_2_ planar structure. All hydrogens except the one pinched between two sulfurs are omitted.

While one might have expected the dangling thiolates in the 2 : 1 complex **[Ni_2_–Fe]^+^** to act as a pendant base, in fact this complex does not show any catalytic activity in the presence of strong acid, HBF_4_·Et_2_O, within the solvent potential window. A computational study, summarized in Fig. 8, reveals that while reduction still occurs on the Fe(NO)_2_ unit, the protonation process is diverted from the Fe(NO)_2_ unit. In this 2 : 1 complex, the computations show that only one thiolate from each NiN_2_S_2_ binds to Fe(NO)_2_, while the other thiolate, is “free” to interact with other electron acceptors; thus it may also be protonated, even before the reduction of the {Fe(NO)_2_}^9^ unit occurs, see Table S9,† and shifts the its potential, which is supported by experiment, *vide supra*.

According to the computations, in the reduced **[Ni_2_–Fe]^0^** the “free” thiolate competes with the reduced {Fe(NO)_2_}^10^ unit for the incoming proton (Fig. 8A); in addition, by rotation around an Fe–S bond, the two NiN_2_S_2_ ligands may orient their “free” thiolate sulfurs to pinch the proton, *i.e.*, consequently forming a strong hydrogen bond (Fig. 8A and B). Spectroscopic evidence supports protonation on S even before reduction, *i.e.*, in **[Ni_2_–Fe]^+^**, Fig. S27.† Two geometries of the pinched proton by two “free” thiolates, **[Ni_2_–Fe–SHS–1]^+^** and **[Ni_2_–Fe–SHS–2]^+^** can be achieved by either translating or rotating one NiN_2_S_2_ unit of **[Ni_2_–Fe]**, respectively. Precedent in Dubois' Ni(P_2_N_2_)_2_ catalysts,^51^ a proton pinched between two amine N bases is relatively stable; in our case, the pinched proton is even more stable than a hydride on Fe(NO)_2_ (Fig. 8A). However, the mechanistic clue from the **[Ni–Fe]** complex^50^ indicates the requirement for a proton to be reduced into a hydride, by {Fe(NO)_2_}^10^, before the H_2_ can be produced by the proton–hydride coupling mechanism. Therefore, the formation of a stable pinched proton likely prevents the generation of the hydride and cuts off the catalytic cycle. The thiolate already bound to Fe(NO)_2_ also helps stabilize the proton on a “free” thiolate, to a smaller extent, with the example of **[Ni_2_–Fe–SHS–3]^+^** (Fig. 8A).

## Conclusions

**Fig. 9:**
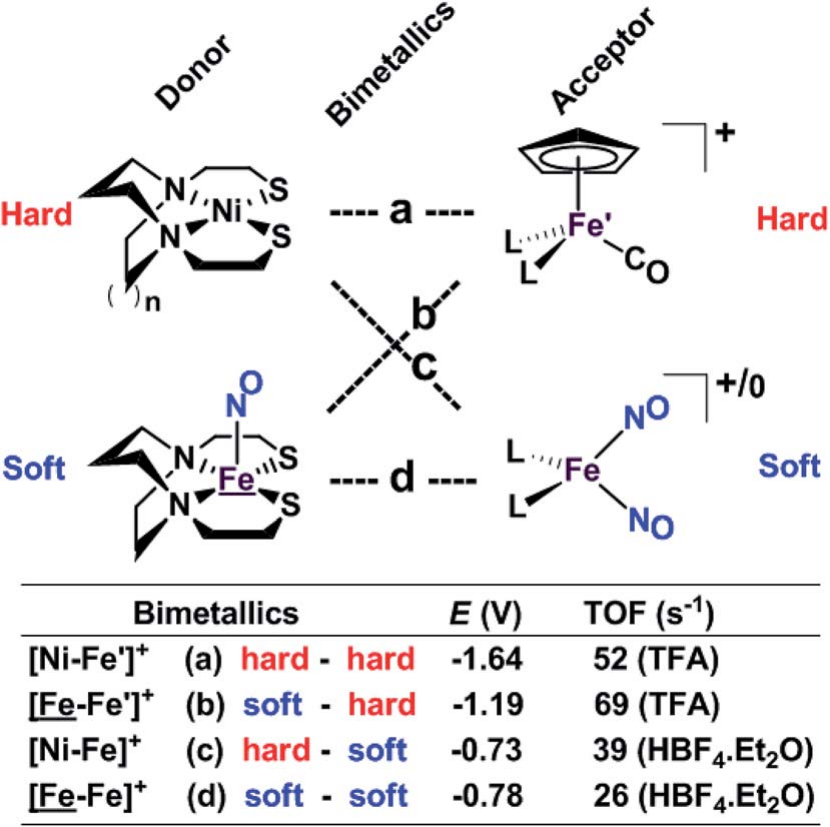
A comparative schematic for a matrix of bimetallic electrocatalysts containing hard/soft donor/acceptor units.

Our collection of hydrogen evolution reaction catalysts is summarized in Fig. 9. While the small differences in donor units and acceptor units do not influence the overall structures of the S-bridged bimetallics; all have butterfly-like [M(μ-SR)_2_Fe] core and the potential for opening up sites for proton addition *via* the hemi-lability of the metallothiolate donors. Nevertheless, demonstrable and explicable differences are seen in their catalytic performances as indicated by catalytic potential, required acid strength, and TOF.

Analogous to the HSAB (Hard and Soft (Lewis) Acids and Bases) concept, we offer an electronic parallel, “soft *vs.* hard donor/receiver units”, in this case directed towards the number of NO ligands in the bimetallics ranging from 0 to 3, with increasing flexibility (*i.e.,* soft) of electronic structure within each unit. The non-innocence of the NO ligand confers electron uptake at milder potentials, which we have seen in both the donor units and acceptor units. Thus the incorporation of NO ligands on the acceptor units, the ‘hard–soft’ and ‘soft–soft’ electrocatalysts lead to energetically more accessible catalytic current, however, at the cost of a stronger acid and a lower TOF in comparison to the bimetallics with hard acceptor units.

While these electrocatalysts are only moderately efficient for H_2_ production, they are well-behaved and have demonstrated reproducibility. Two of the catalysts, c and d, with soft receivers, are isolated and crystallized in both oxidized and reduced forms at ambient conditions lending confidence to the presumed catalytic cycle.

Features in the electrochemical scans may be reasonably ascribed to protonation products whose identities are further described by computational chemistry. The resulting computational mechanisms identify key features that may guide future synthetic targets. For example, the hemi-lability of the S-donors may be optimized by steric constraints; the usefulness of the Fe(NO)_2_ unit as electron depot and protonation site with low redox potential, should encourage explorations with other redox-active, soft acceptors. The computations also suggest a mechanistic paradigm of heterolytic H^–^/H^+^, hydride–proton, coupling for bimetallics **a**, **b** and **c** from the chart, and reductive elimination from **d** arising in the soft–soft construct. Such a supposition derives from extreme electron delocalization in the trinitrosylated 
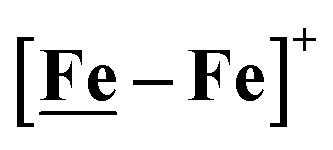
 complex and argues that suitably constructed first row, bimetallic complexes may take on two-electron processes that emulate noble metals.

## Conflicts of interest

There are no conflicts of interest to declare.

## Supplementary Material

Supplementary information

Crystal structure data
