# Supplementary material for: A matrix of heterobimetallic complexes for interrogation of hydrogen evolution reaction electrocatalysts
Source: Chem Sci. 2017 Oct 12;8(12):8291–300. doi: 10.1039/c7sc03378h (PMC5858031; doi:10.1039/c7sc03378h)
Supplement: Supplementary file 1 [file SC-008-C7SC03378H-s001.pdf]

Electronic Supplementary Material (ESI) for Chemical Science.
This journal is © The Royal Society of Chemistry 2017

Supplemental Information

A Matrix of Heterobimetallic Complexes for Interrogation of Hydrogen Evolution Reaction Electrocatalysts

Pokhraj Ghosh,† Shengda Ding,† Rachel B. Chupik,† Manuel Quiroz,† Chung-Hung Hsieh,‡,† Nattami Bhuvanesh,† Michael B. Hall† and Marcetta Y. Darensbourg†*

†Department of Chemistry, Texas A & M University, College Station, TX 77843, United States

‡Department of Chemistry, Tamkang University, New Taipei City, Taiwan 25157

marcetta@chem.tamu.edu

Experimental Section………………………………………………………………………..……4

Preparation of Compounds

Calculation of Magnetic Susceptibility using Evans' Method

Figure S1: ^{19}F NMR Spectrum of **[Ni-Fe]**$^+$ at 22.5 °C using a 500 MHz NMR under Ar referenced to $C_6H_5CF_3$ at -63.7200 ppm. ……………………………………………………………………………7

Figure S2: ^{19}F NMR Spectrum of **[Ni$_2$-Fe]**$^+$ at 20.5 °C using a 500 MHz NMR under Ar referenced to $C_6H_5CF_3$ at -63.7200 ppm. ……………………………………………………………………………8

Electrochemistry

Determination of Overpotential:

Figure S3. Overalay of cyclic voltammograms of **[Ni$_2$-Fe$_2$]**$^{2+}$ (blue trace), **[Ni$_2$-Fe$_2$]**$^{2+}$ with 40 equivalents of $HBF_4.Et_2O$ (red trace) and 50 equivalents of $HBF_4.Et_2O$ (only) without catalyst (green trace). Illustrated herein is the graphical representation for the calculation of $E_{cat/2}$, net catalytic current (i_{cat}-i_{HBF4}) and overpotential. Note: equivalents of $HBF_4•Et_2O$ was calculated with respect to the dimeric **[Ni$_2$-Fe$_2$]**$^{2+}$……………………………………………………………………………………..9

Calculation of Turnover Frequency (TOF):

Table S1. Calculation of TOF for **[Ni-Fe]**$^+$ at various concentrations of $HBF_4.Et_2O$ in CH_2Cl_2 at scan rate of 0.2 V/s……………………………………………………………………………………………10

Table S2. Calculation of TOF for **[<u>Fe</u>-Fe]**$^+$ at various concentrations of $HBF_4.Et_2O$ in CH_2Cl_2 at scan rate of 0.2 V/s……………………………………………………………………………………………10

Experimental setup for bulk electrolysis and gas chromatography:

Figure S4. Charge passed (in coulombs) in 60 min for the bulk electrolysis of **[Ni-Fe]**$^+$ in presence of 100 mM $HBF_4.Et_2O$ in CH_2Cl_2 at -1.12 V. ……………………………...……………………………..…12

Figure S5. Charge passed (in coulombs) in 60 min for the bulk electrolysis of **[Fe-Fe]**$^{+}$ in presence of 100 mM $HBF_4.Et_2O$ in CH_2Cl_2 at -1.12 V. ……………………………………...………………………12

Table S3. Coulombs passed and Faradaic efficiency from three bulk electrolysis experiments of 100 mM $HBF_4.Et_2O$ (free acid) in CH_2Cl_2 at -1.12 V for 60 min. ………………………………………………13

Table S4. Coulombs passed and corrected TON from two bulk electrolysis experiments of 2mM **[Ni-Fe]**$^{+}$ and 100 mM $HBF_4.Et_2O$ in CH_2Cl_2 at -1.12 V for 60 min. …………………………………………....…13

Table S5. Coulombs passed and corrected TON from two bulk electrolysis experiments of 2mM **[Fe-Fe]**$^{+}$ and 100 mM $HBF_4.Et_2O$ in CH_2Cl_2 at -1.12 V for 60 min. ……………………………………………14

Gas Chromatograms:

Figure S6. Gas chromatogram from bulk electrolysis experiment 1 at -1.12 V with 100 mM $HBF_4.Et_2O$ for 60 min in CH_2Cl_2……………………………………………………..………………………14

Figure S7. Gas chromatogram from bulk electrolysis experiment 2 at -1.12 V with 100 mM $HBF_4.Et_2O$ for 60 min in CH_2Cl_2……………………………………………………..………………………15

Figure S8. Gas chromatogram from bulk electrolysis experiment 3 at -1.12 V with 100 mM $HBF_4.Et_2O$ for 60 min in CH_2Cl_2. ……………………………………………………………………..........15

Figure S9. Gas chromatogram from bulk electrolysis experiment 1 at -1.12 V with 2mM **[Ni-Fe]**$^{+}$ and 100 mM $HBF_4.Et_2O$ for 60 min in CH_2Cl_2…………………………………………………………16

Figure S10. Gas chromatogram from bulk electrolysis experiment 2 at -1.12 V with 2mM **[Ni-Fe]**$^{+}$ and 100 mM $HBF_4.Et_2O$ for 60 min in CH_2Cl_2…………………………………………………………16

Figure S11. Gas chromatogram from bulk electrolysis experiment 1 at -1.12 V with 2mM **[Fe-Fe]**$^{+}$ and 100 mM $HBF_4.Et_2O$ for 60 min in CH_2Cl_2…………………………………………………………17

Figure S12. Gas chromatogram from bulk electrolysis experiment 2 at -1.12 V with 2mM **[Fe-Fe]**$^{+}$ and 100 mM $HBF_4.Et_2O$ for 60 min in CH_2Cl_2…………………………………………………………17

Cyclic Voltammograms:

Figure S13. Full scan of cyclic voltammogram of 2.0 mM **[Ni-Fe]**^{0} in CH_2Cl_2. The arrow indicates the initial point and direction of scan.………………………………….……………………......…………18

Figure S14. Cyclic voltammogram of 2.0 mM **[Ni-Fe]**^{0} in CH_2Cl_2. The arrow indicates the initial point and direction of scan.……………..…………………..…………………………………..…..........…18

Figure S15. Full scan of cyclic voltammogram of 2.0 mM **[Ni-Fe]**$^{+}$ in CH_2Cl_2. The arrow indicates the initial point and direction of scan……………………….……….................................. …..........…19

Figure S16. Cyclic voltammogram of 2.0 mM **$[Ni_2\text{-}Fe]^+$** in CH_3CN. The arrow indicates the initial point and direction of scan……………………………………………………………………………19

Figure S17. The reduction event, $\{Fe(NO)_2\}^{9/10}$ ($E_{1/2}$ = -0.72 V), of **$[Ni\text{-}Fe]^+$**, recorded at different scan rates in CH_2Cl_2 referenced to $Fc^{0/+}$……………………………………………………………………20

Figure S18. The reduction event, $\{Fe(NO)_2\}^{9/10}$ ($E_{1/2}$ = -0.72 V), of **$[Ni\text{-}Fe]^0$**, recorded at different scan rates in CH_2Cl_2 referenced to $Fc^{0/+}$……………………………………………………………………20

Figure S19. The reduction event, $\{Fe(NO)_2\}^{9/10}$ ($E_{1/2}$ = -0.78 V), of **$[Ni_2\text{-}Fe]^+$**, recorded at different scan rates in CH_3CN referenced to $Fc^{0/+}$……………………………………………………………………21

Figure S20. A) Cyclic voltammogram of **$[Ni_2\text{-}Fe]^+$**, in CH_3CN referenced to $Fc^{0/+}$, in presence of $HBF_4.Et_2O$. Addition of the third equivalent of $HBF_4.Et_2O$ leads to quasi-reversibility of the redox event at $E_{1/2}$ = -0.52 V, with an overall decrease in the anodic current, i_{pa}, as indicated by the green arrow. **B)** Cyclic voltammograms of 2.0 mM CH_2Cl_2 solutions of **$[Ni\text{-}Fe]^0$** in presence of 0 to 10 equivalents of $HBF_4.Et_2O$. **C)** Charge passed (in coulombs) in 480 min (8 h) for the bulk electrolysis of **[Ni-Fe']$^+$** in presence of 50 equivalents of $HBF_4.Et_2O$ (red) and 50 equivalents of TFA only in absence of **[Ni-Fe']$^+$** (blue), in CH_3CN at -1.73 V vs $Fc^{0/+}$. ……………………………………………………21

Figure S21. EPR spectrum of a CH_2Cl_2 solution of **$[Ni\text{-}Fe]^+$** at 295 K (left) and 77 K (right). The black trace is the experimental spectrum and the red trace is the simulation (*Spin Count*). ……………………………………………………………………………………………22

Figure S22. Frozen solution EPR spectrum of a CH_2Cl_2 solution of **$[Ni_2\text{-}Fe]^+$** at 77 K. The black trace is the experimental spectrum and the red trace is the simulation (*Spin Count*). ………………………………23

IR Spectrum:

Figure S23: Stacked IR plots of **$[Ni_2\text{-}Fe]^+$** and **$[Ni_2\text{-}Fe_2]^{2+}$** in CH_2Cl_2 in black and red, respectively. Note: the N_2S_2 ligand used in this compound is bme-daco. ……………………………………………24

Figure S24: Deconvoluted IR spectra of **$[Ni_2\text{-}Fe_2]^{2+}$** or **$[Ni\text{-}Fe]^+$** in CH_2Cl_2 solution using Lorentzian curve fitting. Fitting parameters are shown on the right. OriginPro8 software was used for fitting. ………24

Figure S25: IR plot of **$[Ni\text{-}Fe]^0$** in THF. Note: the N_2S_2 ligand used in this compound is bme-daco.......25

Figure S26. IR spectrum of **$[Ni_2\text{-}Fe]^+$** in CH_2Cl_2. Note: the N_2S_2 ligand used in this compound is bme-dach. ……………………………………………………………………………………25

Figure S27. IR spectrum of **$[Ni_2\text{-}Fe]^+$** in CH_3CN (blue) and in presence of 1 equivalent of $HBF_4{\cdot}Et_2O$. Note: the N_2S_2 ligand used in this compound is bme-dach. ……………………………………...26

Positive-ion ESI Mass Spectrum:

Figure S28. Positive-ion ESI mass spectrum of **$[Ni\text{-}Fe]^+$** in CH_2Cl_2. …………………………………....26

Figure S29. Positive-ion ESI mass spectrum of **$[Ni\text{-}Fe]^0$** in CH_2Cl_2. …………………………………....27

Figure S30. Positive-ion ESI mass spectrum of **[Ni-Fe]^0** in CH_2Cl_2. ……………………………………....27

Crystal Structures:

Table S6. Crystal data and structure refinement for **[Ni-Fe]^0**. 27

Figure S311. Thermal ellipsoid plot at 50% probability for **[Ni-Fe]^0**..29

Table S7. Crystal data and structure refinement for **[Ni$_2$-Fe$_2$]$^{2+}$**..29

Figure S322. Thermal ellipsoid plot at 50% probability for **[Ni$_2$-Fe$_2$]$^{2+}$**. 31

Table S8. Crystal data and structure refinement for **[Ni$_2$-Fe]$^+$**...31

Figure S33. Thermal ellipsoid at 50 % probability for **[Ni$_2$-Fe]$^+$**..33

Methodology for computation study:

Table S9. The protonation preference on **[Ni$_2$-Fe]$^+$** and **[Ni$_2$-Fe]**...34

Computation Coordinates...34

References …………………………………………………………………………………......48

Experimental Section

General Materials and Techniques. All air sensitive reactions were carried out on a double-manifold Schlenk vacuum line under $N_2$and the compounds were stored in a glovebox withAr atmosphere. Dichloromethane, acetonitrile, methanol, diethylether, hexane, pentane and toluene were freshly purified by an MBraun Manual Solvent Purification System packed with Alcoa F200 activated alumina desiccant. Tetrahydrofuran (THF) was distilled by refluxing over sodium using benzophenone as indicator. The known complexes of NiN_2S_2, where N_2S_2 is either bme-daco or bme-dach (bme-daco = *N*,*N*-bis(2-mercaptoethyl)-1,5-diazacyclooctane[1] and bme-dach = *N*,*N*-bis(2-mercaptoethyl)-1,5-diazacycloheptane[2]), Ni(bme-daco)[1], Ni(bme-dach),[2] $[Fe(CO)_3(NO)]^-Na^+$(18-C-6)[3](where 18-C-6 is the crown ether) and $Fe(CO)_2(NO)_2$[4] were synthesized by published procedures. The following materials were of reagent grade and were used as purchased from Sigma–Aldrich: [n-Bu_4N][PF_6], $AgBF_4$, HPLC-grade acetonitrile, while reagent grade [NO]BF_4, cobaltocene and HBF_4.Et_2O were purchased from Alfa.

Physical Measurements. A Bruker Tensor 37 Fourier transform IR (FTIR) spectrometer was used to record solution IR spectra using a 0.2 mm path length CaF_2 cell. Mass spectrometry (ESI-MS) was performed at the Laboratory for Biological Mass Spectrometry and at Dr. David Russell's laboratory at Texas A&M University. The ^{19}F NMR spectra were recorded on an Inova 500 MHz superconducting NMR instrument. The ^{19}F NMR spectra were referenced to $C_6H_5CF_3$ at -63.7200 ppm. X-Band Bruker300Espectrometer was used to measure CW EPR spectrum at 9.3701 GHz frequency at 298 or 77 K. Spin Count developed by Prof. M. P. Hendrich of Carnegie Mellon University was used to simulate the spectra. OriginPro 8 SR4 v8.0951 (B951) developed by OriginLab Corporation was used for deconvolution of IR spectra.

X-ray Crystal Structure Determination.

For all complexes, X-ray data at low temperature (150 K) was obtained using a Bruker Apex-II CCD based diffractometer (Texas A&M University) (Mo sealed X-ray tube, K_α = 0.71073 Å). Mineral oil coated crystal sample was affixed to a nylon loop which was placed under a stream N_2 at low temperature for data collection. Systematic absences and intensity statistics were used to determine space groups, and direct methods were used to solve structures which were refined by full-matrix least-squares on F_2. Anisotropic thermal parameters were used to refine non-hydrogen atoms. Anisotropic displacement parameters were used for all non-hydrogen atoms. Placed at idealized positions H atoms were refined with fixed isotropic displacement parameters. The following programs were used: APEX2 for data collection;[5] SAINT for data reduction;[6] SADABS for absorption correction;[7] SHELXTL for cell refinement; [7] SHELXS-97 for structure solutions; [7] and SHELXL-97for structure refinement.[7] X-Seed Version 2.0.was used for final data presentation and structure plots.[8] Crystallographic data for the complexes **[Ni-Fe]^0**, **[Ni$_2$-Fe$_2$]$^{2+}$** and **[Ni$_2$-Fe]$^+$** were deposited in the Cambridge Crystallographic Data Centre. The following CCDC numbers were assigned to them: **[Ni-Fe]^0** (CCDC 1045461), **[Ni$_2$-Fe$_2$]$^{2+}$** (CCDC 1045460) and **[Ni$_2$-Fe]$^+$** (CCDC 1565539).

Preparation of Compounds.

Syntheses of [NiN$_2$S$_2$•Fe(NO)$_2$(CO)], [Ni-Fe(CO)]and **[Ni(bme-daco)•Fe(NO)$_2$], [Ni-Fe]^0.** A solution of Ni(bme-daco) (0.29g, 0.10 mmol) in 15 mL THF was anaerobically added to a freshly trapped orange solution of $Fe(CO)_2(NO)_2$[4] (0.11mmol) in 15 mL THF and was stirred for 10 min at room temperature in absence of light. The reaction was monitored by changes in the IR spectrum for appropriate shift of the ν(CO) and ν(NO) stretching frequencies for the formation of **[Ni-Fe(CO)]**. IR (THF, cm^{-1}): ν(CO) 2006; ν(NO) 1734, 1690.**[Ni-Fe]^0** was synthesized upon heating **[Ni-Fe(CO)]** solution at 40 °C for 20 min or by stirring under UV light for 5 to 10 min.

The course of the reaction should be monitored by IR spectrum as overheating or excess irradiation leads to decomposition. Upon completion of the reaction, the resulting brown solution was filtered over dry celite and was partially kept under vacuum to remove excess $Fe(CO)_2(NO)_2$. The concentrated brown THF solution was recrystallized by layering with hexane at – 35 °C to afford brown X-ray quality crystals. IR (THF, cm^{-1}): ν(NO) 1681 (m), 1630 (s). ESI-MS$^+$: *m/z* 405.9870 (Calc. for [M], 405.9731).

Synthesis of $[NiN_2S_2•Fe(NO)_2][BF_4]$, $[Ni\text{-}Fe]^+$ or **$[Ni(bme\text{-}daco)•Fe(NO)_2]_2[BF_4]_2$, $[Ni_2\text{-}Fe_2]^{2+}$.** Reactants Ni(bme-daco) (0.29 g, 0.10 mmol), $[Fe(CO)_3(NO)]^-Na^+(18\text{-}C\text{-}6)$[3] (0.47 g, 0.10 mmol) and $[NO]BF_4$(0.23 g, 0.20 mmol) were stirred in 20 mL CH_2Cl_2 for 5 h under N_2. The reaction was monitored by IR spectrum. Upon completion the purple reaction mixture was concentrated to around ~ 5 mL and was precipitated by adding pentane. The precipitate was washed with diethyl ether (3 x 15 mL) and pentane (2 x 10 mL). The precipitate was redissolved in 10 mL $CH_2Cl_2$and was filtered through dry celite to remove impurities (Yield: 0.29 g, 60 %). Dark purple X-ray quality crystals of **$[Ni_2\text{-}Fe_2]^{2+}$**were grown in CH_2Cl_2/pentane at -35 °C as BF_4^- salt.IR (CH_2Cl_2, cm^{-1}): ν(NO) 1805 (m), 1794 (s), 1749 (m), 1732 (s). ESI-MS$^+$: *m/z* 405.9737 (Calc. for $[M]^+$, 405.9731).

Synthesis of $[(NiN_2S_2)_2•Fe(NO)_2][BF_4]$, $[Ni_2\text{-}Fe]^+$. In a manner similar to that of above **$[Ni_2\text{-}Fe]^+$**was synthesized by stirring reactants Ni(bme-daco) (0.56 g, 0.20 mmol) or Ni(bme-dach) (0.56 g, 0.20 mmol), $[Fe(CO)_3(NO)]^-Na^+(18\text{-}C\text{-}6)$[3] (0.47 g, 0.10 mmol) and $[NO]BF_4$(0.23 g, 0.20 mmol) in 20 mL CH_2Cl_2 for 5 h under N_2. Recrystallization in CH_2Cl_2/hexane at -35 °C afforded dark brown X-ray quality crystals of **$[Ni_2\text{-}Fe]^+$** as BF_4^- salt (Yield: 0.40 g, ~ 55 %). IR (CH_2Cl_2, cm^{-1}): ν(NO) 1790(m), 1736 (s) for complex having bme-dach ligand and ν(NO) 1789 (m), 1736 (s) for complex having bme-daco ligand. ESI-MS$^+$ (for complex having bme-dach ligand): *m/z* 667.9484 (Calc. for $[M]^+$, 667.9839.

Calculation of Magnetic Susceptibility using Evans Method

The effective magnetic moment (μ_{eff}) of a compound is calculated according to following equation:

$\mu_{eff} = \chi_p T = (1/8)[n(n+2)]$

χ_p= paramagnetic susceptibility

T = absolute temperature

n =number of unpaired electrons

The experimentally measured magnetic susceptibility (χ_{expt}) is the sum of χ_p and χ_D, where χ_D is the diamagnetic susceptibility. χ_D is independent of temperature with a negative magnitude, and is a property arising from all atoms in the compound.

$$\chi_{expt} = \chi_p + \chi_D$$

Thus, the diamagnetic susceptibility should be taken into account in order to calculate μ_{eff}.[9]

The ^{19}F NMR of **[Ni-Fe]**$^+$and **[Ni$_2$-Fe]**$^+$was measured in a 500 MHz NMR machine, using 9 mg and 12 mg of the compounds, respectively, with $C_6H_5CF_3$ as the standard, at 22.5 °C and 20.5 °C. A coaxial NMR tube was used for this purpose. The outer tube consisted of the Ni-Fe complex dissolved in 0.5 mL of CD_2Cl_2 and 1.245 µL of $C_6H_5CF_3$ while the inner tube contained only 0.5 mL of CD_2Cl_2 and 1.245 µL of $C_6H_5CF_3$. The ^{19}F NMR spectra of **[Ni-Fe]**$^+$ and **[Ni$_2$-Fe]**$^+$are shown in Figure S1 and S2, respectively.

The calculated χ_D of **[Ni-Fe]**$^+$ and **[Ni$_2$-Fe]**$^+$ were-0.0002393 and -0.0003881, respectively, which are close to [–(mol.wt.)/2]/1000000.[9]

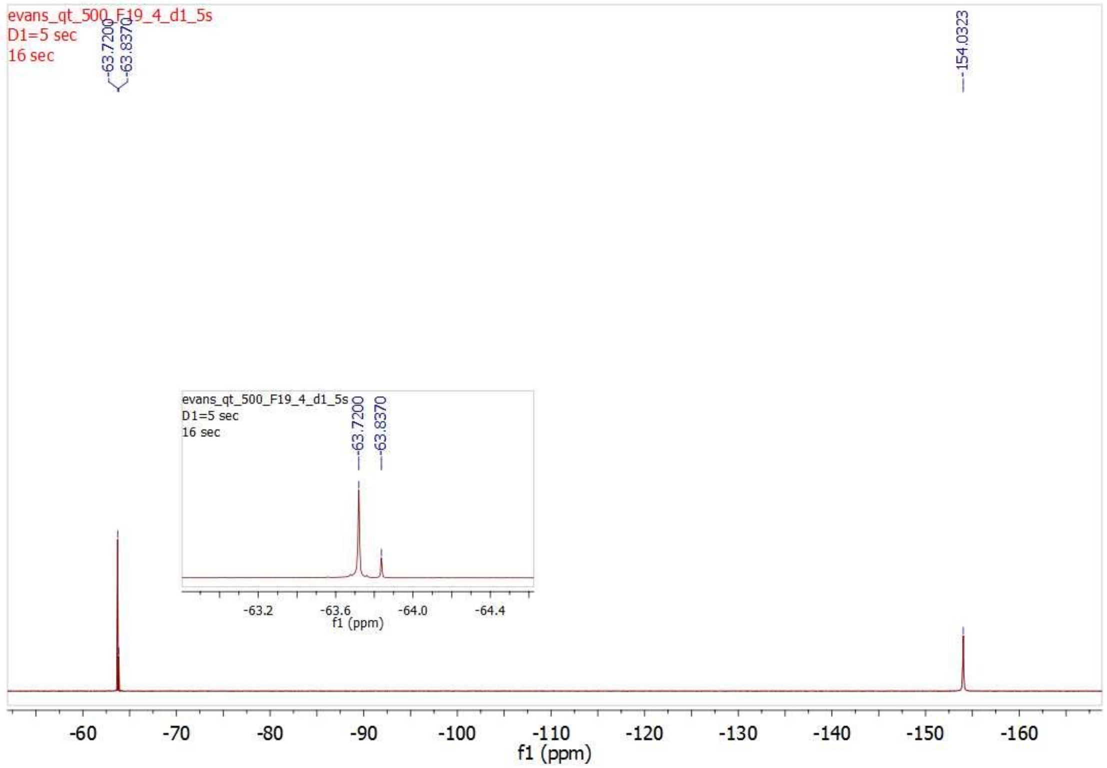

Figure S1: ^{19}F NMR Spectrum of **[Ni-Fe]**$^+$ at 22.5 °Cusing a 500 MHz NMR under Ar referenced to $C_6H_5CF_3$ at -63.7200 ppm.

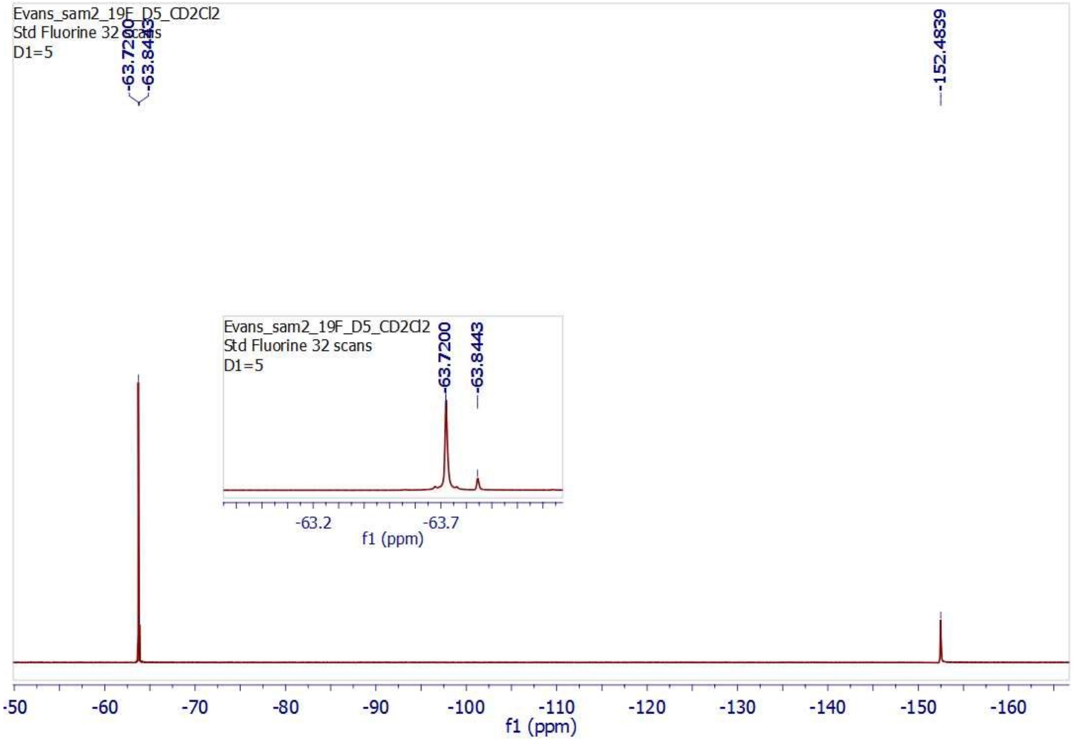

Figure S2: ^{19}F NMR Spectrum of **[Ni_2-Fe]$^+$** at 20.5 °C using a 500 MHz NMR under Ar referenced to $C_6H_5CF_3$ at -63.7200 ppm.

Electrochemistry

Determination of Overpotential:

Overpotentials were calculated by using methods determined by Appel and Helm for complexes **[Ni_2-Fe_2]$^{2+}$** and **[Fe-Fe]$^+$**.[10-14] The difference between the catalytic half wave potential ($E_{cat/2}$) and the thermodynamic potential (E_{H^+}) is known as the overpotential. Using 100 mM tetrafluoroboric acid ($HBF_4.Et_2O$) in dichloromethane, the thermodynamic potential (E_{H^+}) utilized was -0.26 V (in CH_3CN)[13, 14] (vs $Fc^{0/+}$ = 0.0 V). The catalytic half wave potential ($E_{cat/2}$) corresponds to half the value of the potential where the catalytic current (i_{cat}) is located. Figure S3 shows an example for the **[Ni_2-Fe_2]$^{2+}$** as an illustration of the mentioned parameters.

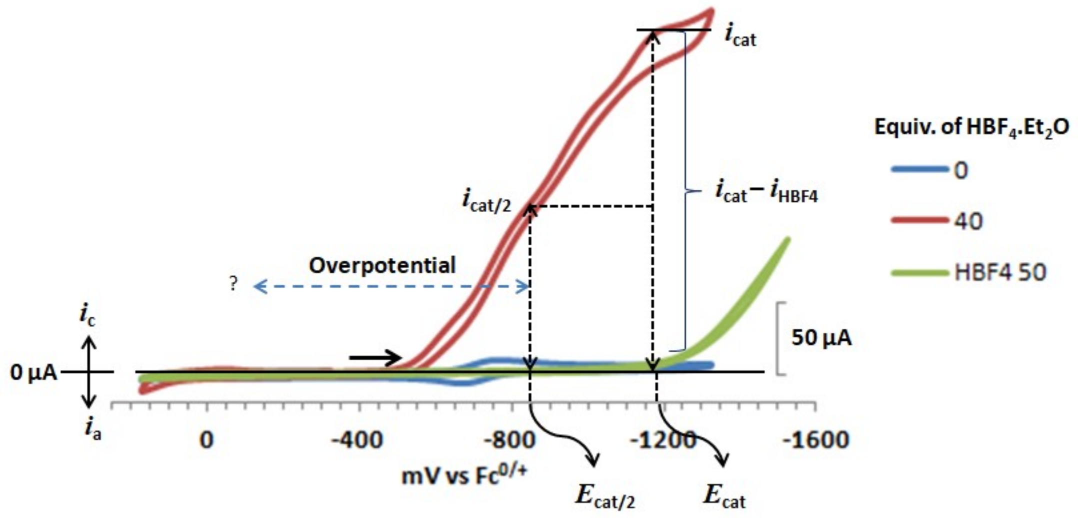

Figure S3.Overalay of cyclic voltammograms of **[Ni_2-Fe_2]$^{2+}$** (or **[Ni-Fe]$^+$**,blue trace), **[Ni_2-Fe_2]$^{2+}$** with 40 equivalents of $HBF_4.Et_2O$ (or **[Ni-Fe]$^+$**,red trace) and 50 equivalents of $HBF_4.Et_2O$ (only) without catalyst (green trace). Illustrated herein is the graphical representation for the calculation of $E_{cat/2}$, net catalytic current (i_{cat}-i_{HBF4}) and overpotential.Note: equivalents of HBF_4•Et_2O was calculated with respect to the dimeric [**Ni_2-Fe_2**]$^{2+}$.

Calculation of Turnover Frequency (TOF):

The Darensbourg group recently showed the calculation of TOF frequency according to a modified equation that accounts for the contribution ofthe background acid from the catalytic current, at E_{cat}, by subtracting i_{HBF4} fromi_{cat} (Eq. 2).[15, 16] The general form of the equation is shown in (Eq. 1).[10, 11]

$$TOF = 1.94\ (V^{-1}) *v\ (Vs^{-1}) * [i_{cat}/i_p]^2 \text{(Eq. 1)}$$

$$TOF = 1.94\ (V^{-1}) *v\ (Vs^{-1}) * [(i_{cat}-i_{HBF4})/i_p]^2 \quad \text{(Eq. 2)}$$

v = scan rate
i_{cat}= total catalytic current measured upon adding $HBF_4.Et_2O$ to the catalyst
i_{HBF4}= background current measured from $HBF_4.Et_2O$ at E_{cat}
i_p= current measured fromthe catalyst in absence of acid
i_{cat}-i_{HBF4} = corrected catalytic current response

$HBF_4.Et_2O$ was sequentially added to a 2 mM solution of **[Ni-Fe]$^+$** or **[<u>Fe</u>-Fe]$^+$**, in CH_2Cl_2, until the catalytic current was fairly constant as shown in Tables S1 and S2, respectively.The TOF frequency of **[<u>Fe</u>-Fe]$^+$**was calculated under similar experimental setup for apt comparison.[17]

Note: The increase in cathodic current for the free acid, $HBF_4.Et_2O$ (only), was practically negligible upon sequential addition of the acidat E_{cat}. Hence the value of i_{HBF4}, employed for TOF calculation, was the cathodic currentat 50 equivalents of the acid; i.e. $i_{HBF4} = 0.055 \times 10^{-4}$ A.

Table S1. Calculation of TOF for **[Ni-Fe]$^+$** at various concentrations of $HBF_4.Et_2O$ in CH_2Cl_2 at scan rate of 0.2 V/s. The $\{Fe(NO)_2\}^{9/10}$ redox event at -0.73 V, i.e. the onset of the catalytic event, was considered for calculating i_p.

Acid (equiv.)	i_{cat} * 10^4 (A)	i_{cat}-i_{HBF4} (mA)	TOF (s^{-1})
0	0	---	0.000
24	1.689	1.633	17.08
26	1.811	1.755	19.72
28	1.858	1.802	20.79
31	1.903	1.847	21.84
37	2.145	2.089	27.94
41	2.337	2.281	33.31
49	2.458	2.402	36.94
61	2.485	2.429	37.77
70	2.529	2.473	39.16
79	2.545	2.489	39.66

Table S2. Calculation of TOF for **[Fe-Fe]$^+$** at various concentrations of $HBF_4.Et_2O$ in CH_2Cl_2 at scan rate of 0.2 V/s. The $\{Fe(NO)_2\}^{9/10}$ redox event at -0.78 V, i.e. the onset of the catalytic event, was considered for calculating i_p

Acid (equiv.)	i_{cat} * 10^4 (A)	i_{cat}-i_{TFA} (mA)	TOF (s^{-1})
0	0	---	0.000
9	1.103	1.047	7.73
15	1.298	1.242	10.87
22	1.397	1.341	12.67

42	1.763	1.707	20.53
54	1.840	1.784	22.43
66	1.931	1.875	24.77
72	1.979	1.923	26.06
84	2.003	1.947	26.70

Experimental setup for bulk electrolysis and gas chromatography:

The apparatus used for bulk electrolysis experiments consisted of a three-neck truncated conical shaped flask equipped with an outlet port/gas inlet which was custom made in the A&M chemistry glass shop. In the necks of the cell a 3 mm glassy carbon working electrode, a Ni-Cr-coiled wire counter electrode, and a $Ag/AgNO_3$ reference electrode were inserted. The counter electrode was made by placing Ni-Cr-coiled wire in glass tube with a medium glass frit. The reference electrode was a glass tube with a Ag wire submerged in a 1 mM CH_3CN solution of $AgNO_3$ which was separated from the main solution by a Vycor frit. The electrolyte solution in the electrochemical cell contained 10 mL of 0.1 M [n-Bu_4N][PF_6] in CH_2Cl_2 and was degassed by purgingAr/N_2. The cell containing $2x10^{-5}$ mol of the desired catalyst was treated with 50 equivalents or 100 mM of $HBF_4.Et_2O$. After the addition of acid, catalytic activity was first determined by cyclic voltammogram. 1mL of methane was used as an internal standard after bulk electrolysis was performed at -1.12 V vs Fc/Fc^+ for 60 min. Note: equivalents of HBF_4•Et_2O was calculated with respect to the 2 mM dimeric [$\mathbf{Ni_2}$-$\mathbf{Fe_2}$]$^{2+}$.

The gasses were identified by gas chromatography using an Agilent Trace 1300 GC equipped with a custom-made 120 cm stainless steel column packed with Carbosieve-II and a thermal conductivity detector. For gas separation, the column was maintained at 200 °C and Ar was used as the carrier gas. The temperature of the detector was set to 250 °C. To quantify the production of H_2after bulk electrolysis, about 200 µL of the headspace gas was withdrawn using a 0.5 mL Valco Precision Sampling Synringe (Series A-2) with a Valco Precision sampling needle; a 5 point side port was attached to the needle. H_2 was quantified by using the internal standard and the relative response factor of H_2. The relationship $\frac{area\ of\ H_2}{area\ of\ CH_4}$ vs $\frac{mL\ of\ H_2}{mL\ of\ CH_4}$ was used to plot the calibration curve. This calibration curve was previously determined by gas chromatograms of various amounts of H_2 vs 1 mL of methane. The linear equation of the calibration curve was $y = 2.9757x + 0.0226$ with R^2 value of 0.9987.[15]

In absence of catalyst, three bulk electrolysis experiments were conducted at -1.12 V in CH_2Cl_2 using 50 equivalents or 100mM $HBF_4.Et_2O$ (free acid) in an Ar/N_2 atmosphere for background H_2 evolution.The average Faradic efficiency for three experiments was 38.69 ± 0.08% while the average charge passed was 0.098 ± 0.009 C. To account for the background H_2, produced by $HBF_4.Et_2O$ (free),it was subtracted from the total H_2 produced from the bulk electrolysis experiment with 2 mM catalyst and 50 equivalents or 100mM $HBF_4.Et_2O$. Figure S4s hows the comparison of charge passed vs time (s) for the bulk electrolysis of 100 mM of background $HBF_4.Et_2O$ (free acid) and **[Ni-Fe]**$^+$in presence of 100 mM of $HBF_4.Et_2O$ in CH_2Cl_2 at -1.12 V. A similar graph is shown for the bulk electrolysis of **[Fe-Fe]**$^+$ under similar

experimental conditions, Figure S5. The gas chromatograms are listed in Figures S6-S12. Atmosphere nitrogen and oxygen, that might have contaminated the needle of the gas-tight syringe prior to the insertion of the head space gas to the gas chromatograph were assigned to be the peaks at retention times~2.27 min. Tables S3-S5 show the faradic efficiency and the corrected TON.

Figure S4. Charge passed (in coulombs) in 60 minutes for the bulk electrolysis of **[Ni-Fe]$^+$** in presence of 100 mM $HBF_4.Et_2O$ in CH_2Cl_2 at -1.12 V.

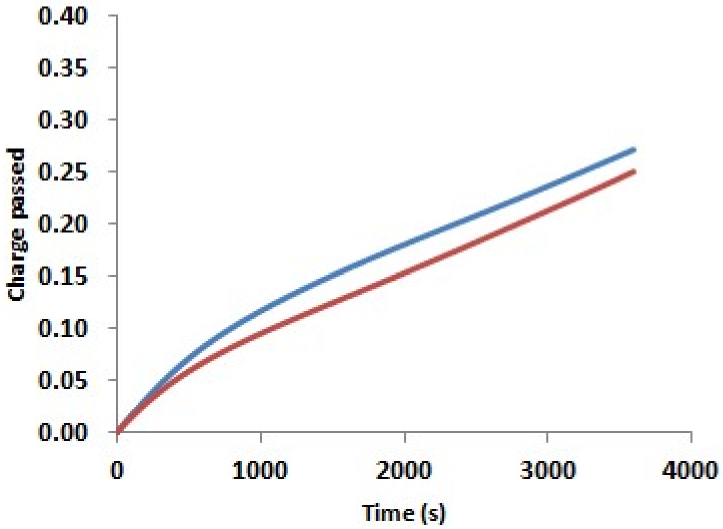

Figure S5. Charge passed (in coulombs) in 60 minutes for the bulk electrolysis of **[Fe-Fe]$^+$** in presence of 100 mM $HBF_4.Et_2O$ in CH_2Cl_2 at -1.12 V.

Table S3. Coulombs passed and Faradaic efficiency from three bulk electrolysis experiments of 100 mM $HBF_4.Et_2O$ (free acid) in CH_2Cl_2 at -1.12 V for 60 min.

Exp.	Coulombs Passed (C)	Area of H_2	Area of CH_4	Ratio: $Area_{H2}/Area_{CH4}$	Theoretical H_2 (mL)	Observed H_2 (mL)	Faradaic Efficiency (%)
1	0.1005	0.0122	0.3271	0.0373	0.0128	0.0049	38.72
2	0.0876	0.0114	0.3320	0.0354	0.0111	0.0043	38.76
3	0.1053	0.0126	0.3218	0.0379	0.0134	0.0051	38.60

Average coulombs passed (rounded to three decimal places) = 0.098± 0.009
Average Faradaic efficiency = 38.69 ± 0.08%

Table S4. Coulombs passed and corrected TON from two bulk electrolysis experiments of 2mM **[Ni-Fe]**$^{+}$ and 100 mMHBF$_4$.Et$_2$O in CH$_2$Cl$_2$ at -1.12 V for 60 min.

Experiment	Charge Passed (C)	Corrected Charge Passed (C)	Faradaic Efficency (%)	TON	Corrected TON
**1**	0.2361	0.1383	66.144	0.061	0.036
**2**	0.2126	0.1148	69.117	0.055	0.030
**Average (rounded to three decimals)**	0.224 ± 0.017	0.125 ± 0.017	67.631 ± 2.102	0.058 ±0.004	0.033 ± 0.004

Table S5. Coulombs passed and corrected TON from two bulk electrolysis experiments of 2mM **[Fe-Fe]**$^{+}$ and 100 mM HBF$_4$.Et$_2$O in CH$_2$Cl$_2$ at -1.12 V for 60 min.

Experiment	Charge passed (C)	Corrected Charge passed (C)	Faradaic Efficency (%)	TON	Corrected TON
**1**	0.2496	0.1518	59.449	0.064	0.039
**2**	0.2722	0.1744	57.593	0.071	0.045

**Average (rounded to three decimals)**	0.261 ± 0.016	0.163 ± 0.016	58.521 ± 1.313	0.068 ± 0.004	0.042 ± 0.004

Note: The TONs reported here are in three decimal places. A precision of three decimal places is unrealistic under experimental conditions; hence for comparison a rounded value would provide more physical sense.

The TONs for the complexes **[Ni-Fe]**$^+$ and **[Fe-Fe]**$^+$ were calculated to be 0.033 and 0.042, respectively. According to the procedures mentioned above the bulk electrolysis was carried out for a period of 1 h in CH_2Cl_2 with 50 equivalents of $HBF_4.Et_2O$ at a constant potential of -1.12 V (vs $Fc^{0/+}$). The TON of **[Ni-Fe']**$^+$ was found to be 6.78 using 50 equivalents of TFA for a period of 8 h. The bulk electrolysis was done at a constant potential of -1.73 V (vs $Fc^{0/+}$) in CH_3CN. The TON of **[Fe-Fe']**$^+$ complex was 0.33 using 50 equivalents of TFA for a period of 0.5 h, at a constant potential of -1.563 V (vs $Fc^{0/+}$) in CH_3CN.[15]

Gas Chromatograms:

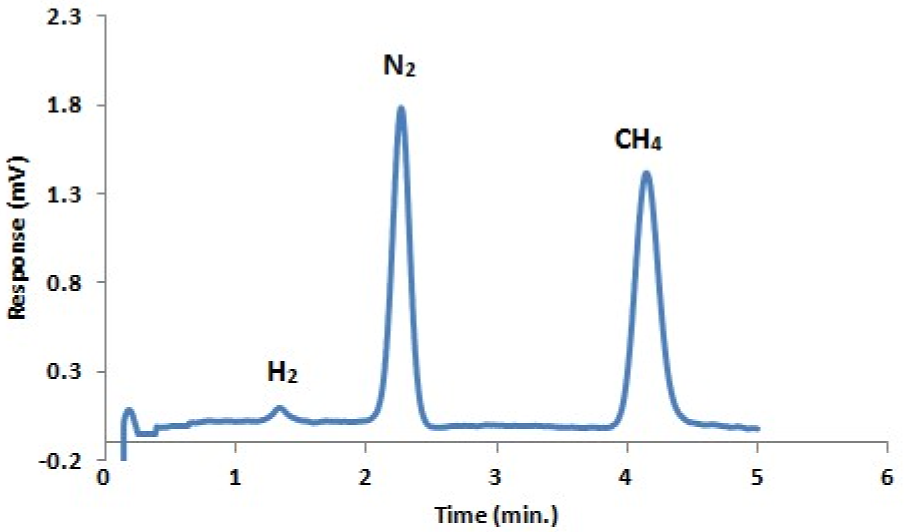

Figure S6. Gas chromatogram from bulk electrolysis experiment 1 at -1.12 V with 100 mM $HBF_4.Et_2O$ for 60 min in CH_2Cl_2.

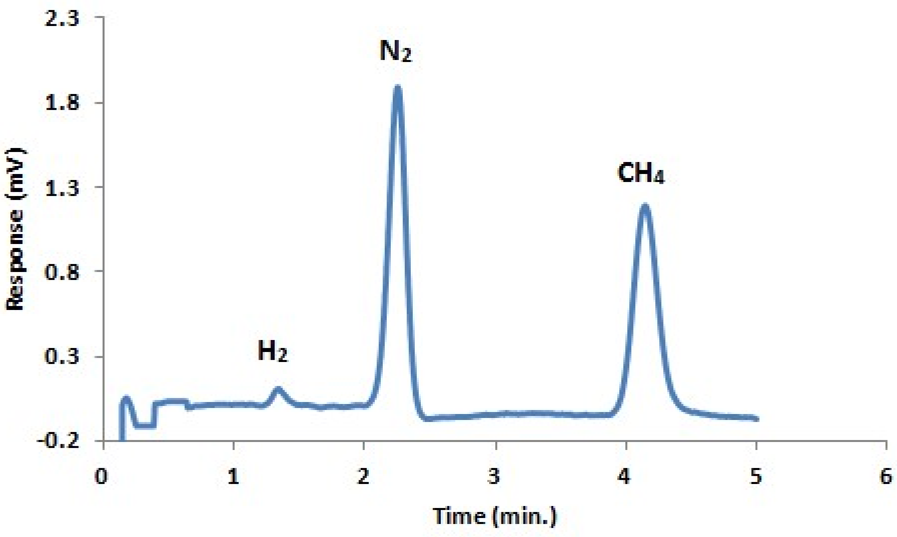

Figure S7. Gas chromatogram from bulk electrolysis experiment 2 at -1.12 V with 100 mM $HBF_4.Et_2O$ for 60 min in CH_2Cl_2.

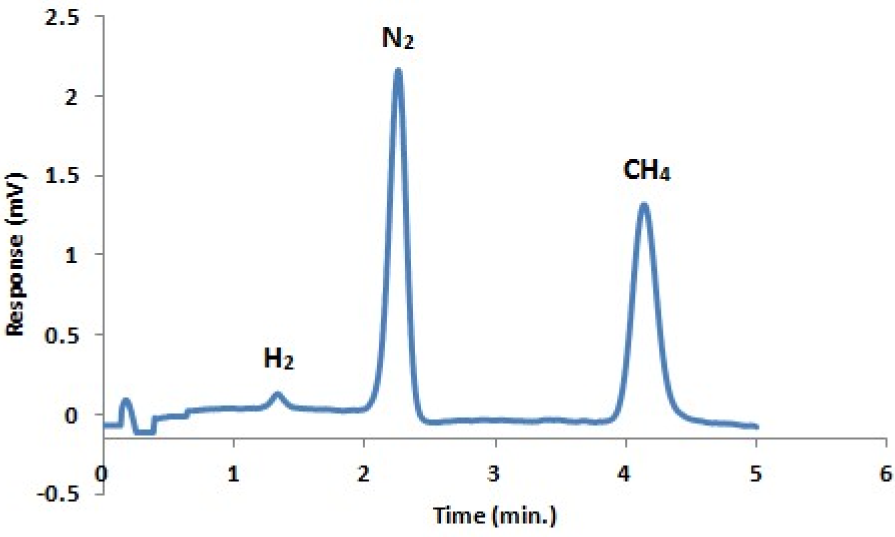

Figure S8. Gas chromatogram from bulk electrolysis experiment 3 at -1.12 V with 100 mM $HBF_4.Et_2O$ for 60 min in CH_2Cl_2.

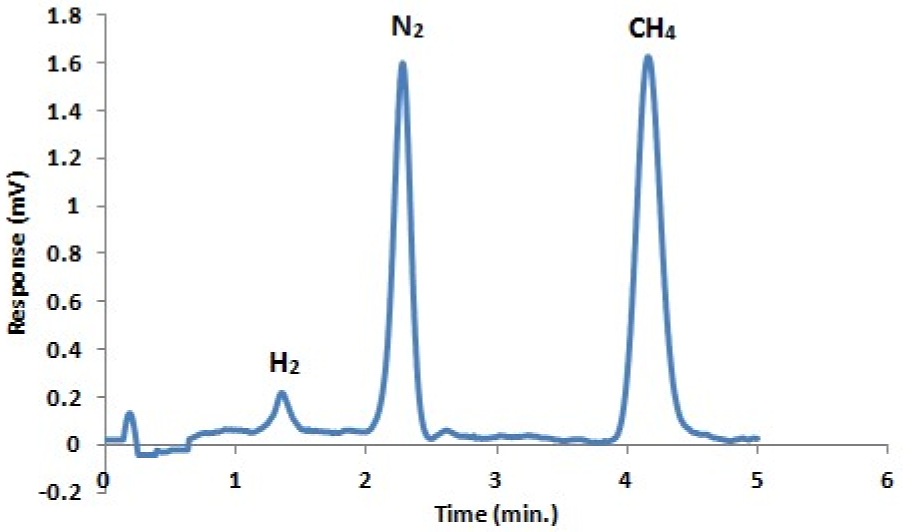

Figure S9. Gas chromatogram from bulk electrolysis experiment 1 at -1.12 V with 2mM **[Ni-Fe]**$^+$ and 100 mM $HBF_4.Et_2O$ for 60 min in CH_2Cl_2.

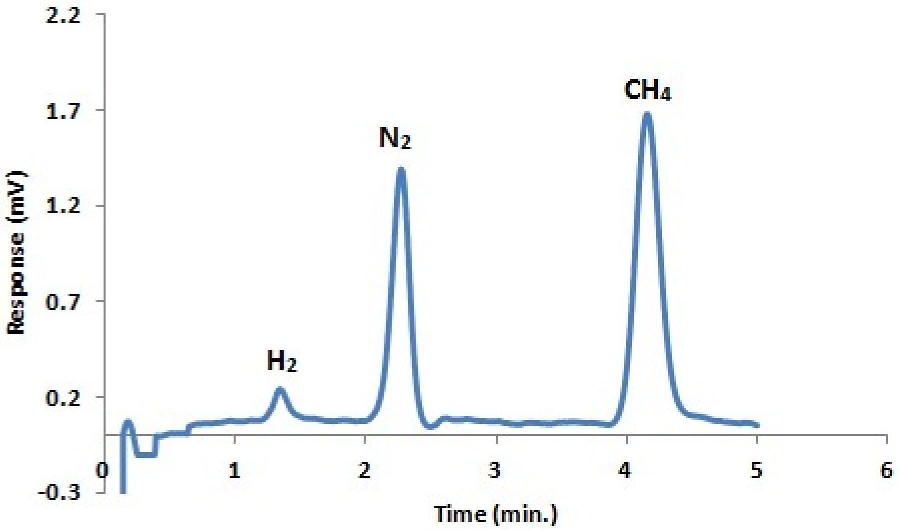

Figure S10. Gas chromatogram from bulk electrolysis experiment 2 at -1.12 V with 2mM **[Ni-Fe]**$^+$ and 100 mM $HBF_4.Et_2O$ for 60 min in CH_2Cl_2.

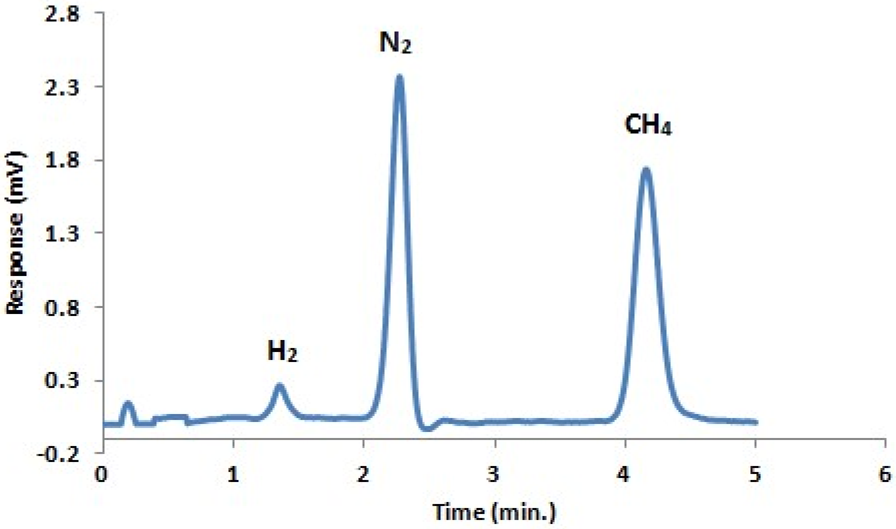

Figure S11. Gas chromatogram from bulk electrolysis experiment 1 at -1.12 V with 2mM **[Fe-Fe]**$^+$ and 100 mM $HBF_4.Et_2O$ for 60 min in CH_2Cl_2.

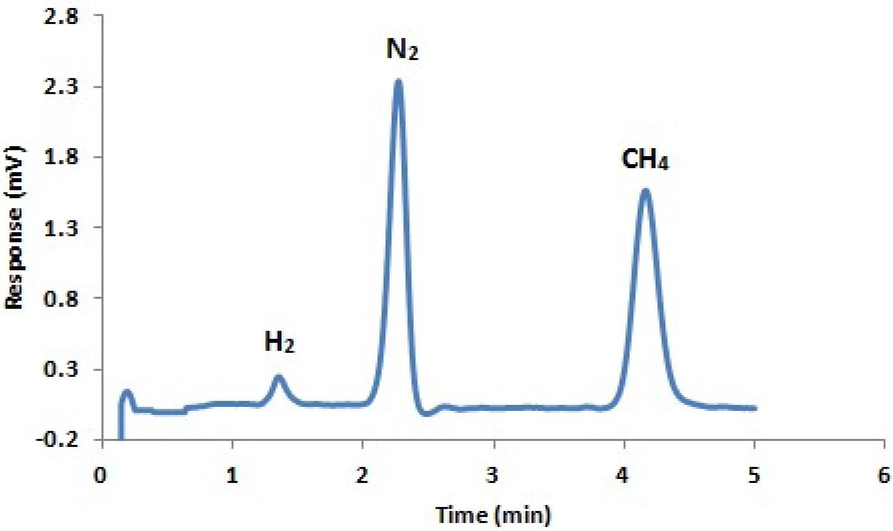

Figure S12. Gas chromatogram from bulk electrolysis experiment 2 at -1.12 V with 2mM **[Fe-Fe]**$^+$ and 100 mM $HBF_4.Et_2O$ for 60 min in CH_2Cl_2.

Cyclic Voltammograms:

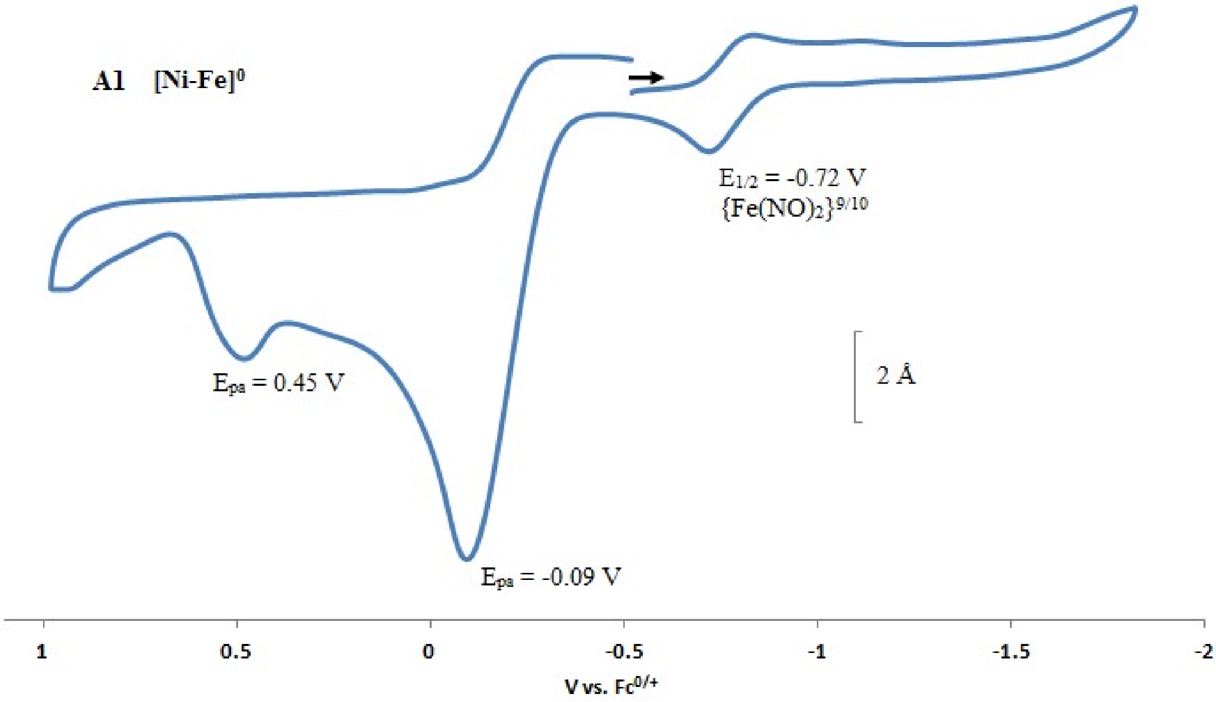

Figure S13. Full scan of cyclic voltammogram of 2.0 mM **[Ni-Fe]^0** in CH_2Cl_2. The arrow indicates the initial point and direction of scan.

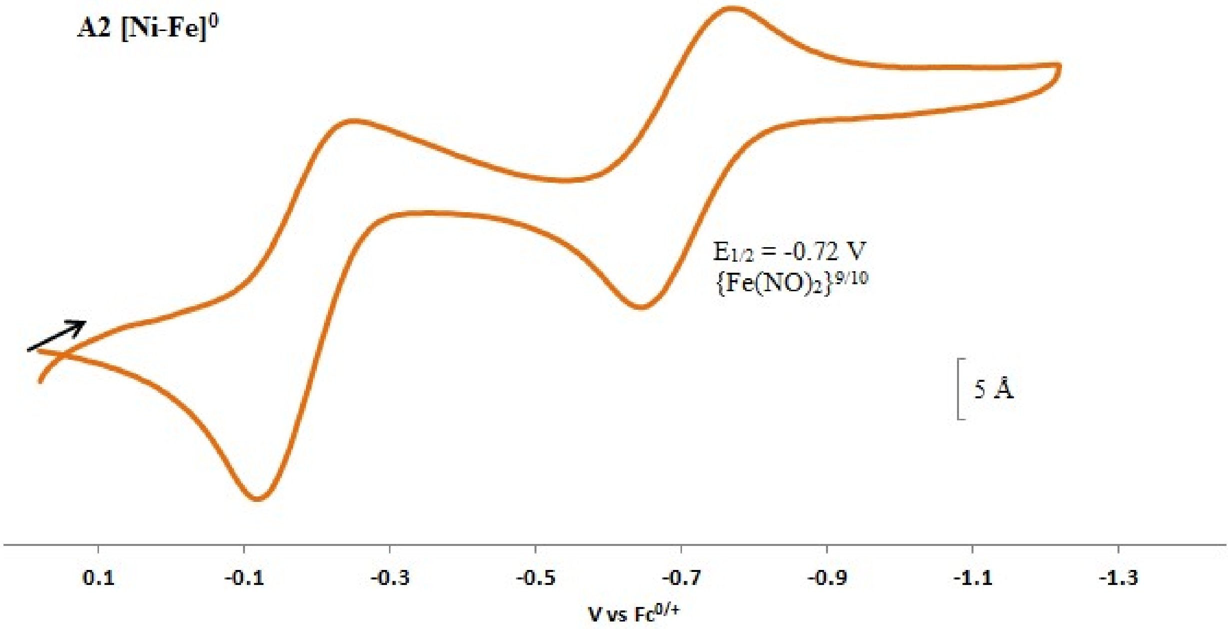

Figure S14. Cyclic voltammogram of 2.0 mM **[Ni-Fe]^0** in CH_2Cl_2. The arrow indicates the initial point and direction of scan.

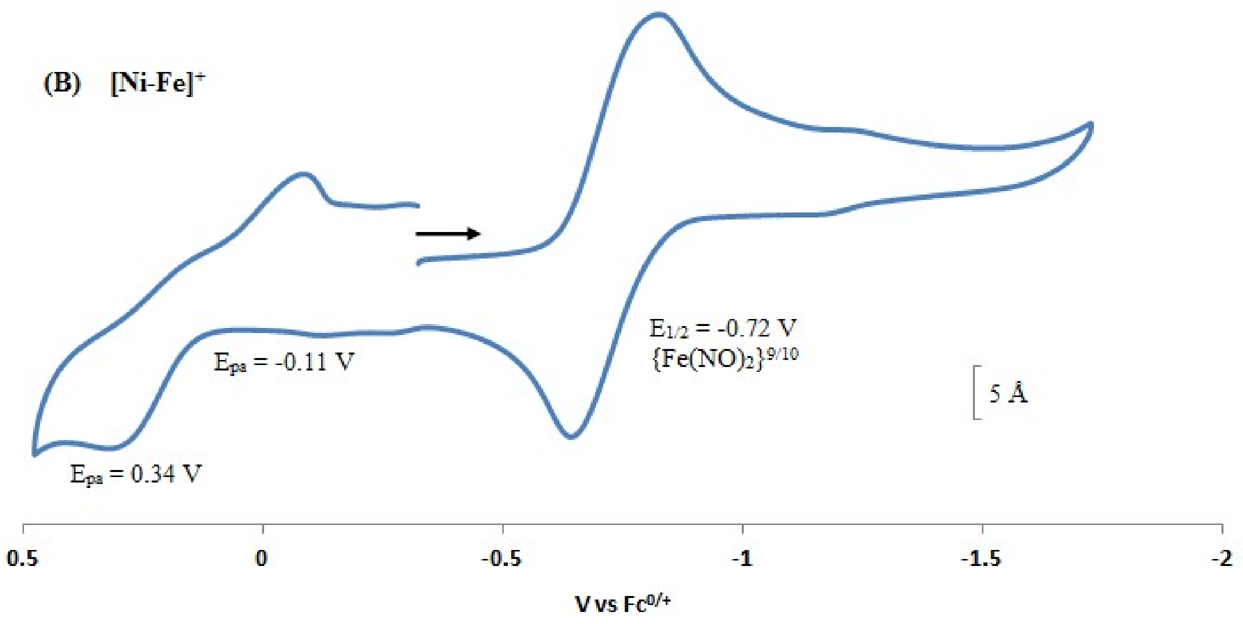

Figure S15. Full scan of cyclic voltammogram of **[Ni-Fe]**$^+$ in CH_2Cl_2 (2 mM **[Ni$_2$-Fe$_2$]**$^{2+}$). The arrow indicates the initial point and direction of scan.

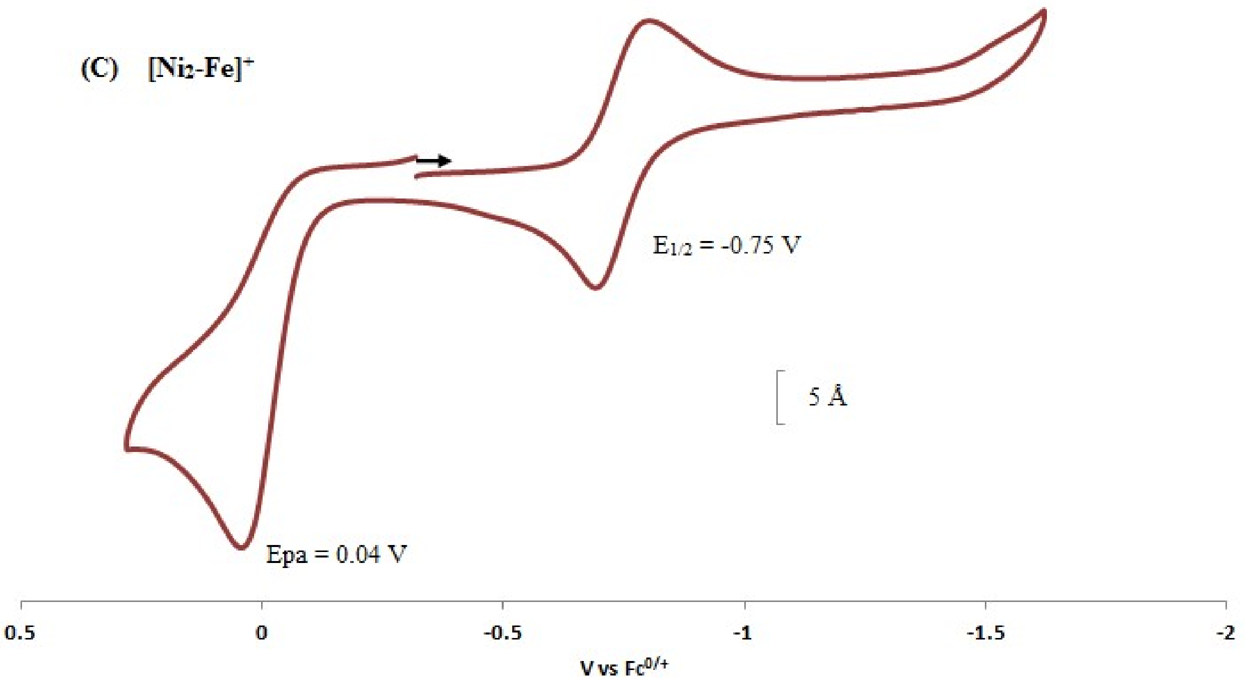

Figure S16. Cyclic voltammogram of 2.0 mM **[Ni$_2$-Fe]**$^+$in CH_3CN. The arrow indicates the initial point and direction of scan. Note: the N_2S_2 ligand used in this compound is bme-dach.

Scan Rates:

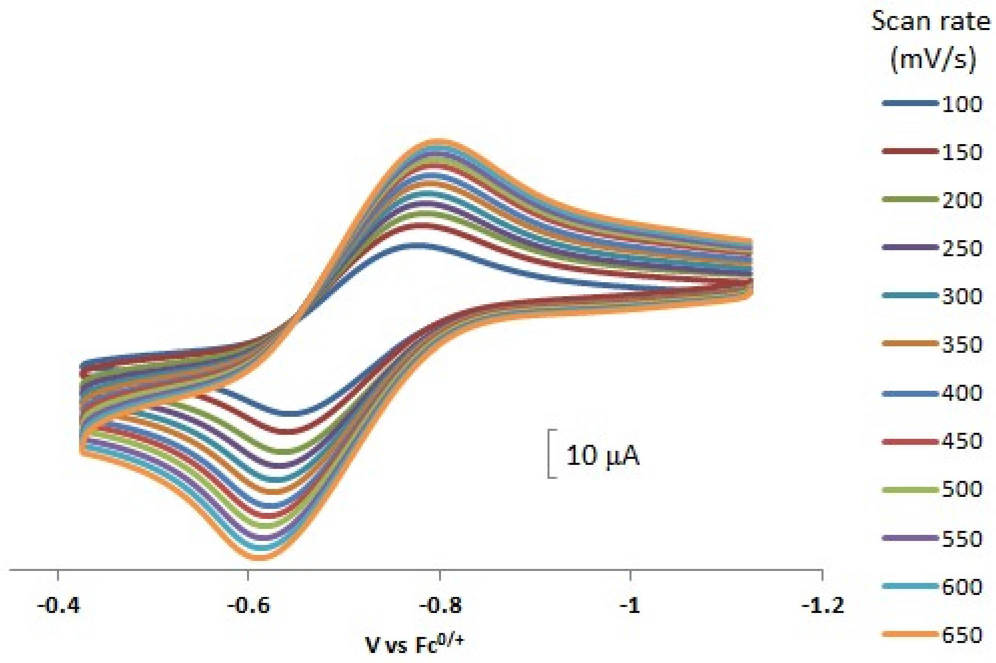

Figure S17. The reduction event, $\{Fe(NO)_2\}^{9/10}$ ($E_{1/2}$ = -0.72 V), of **[Ni-Fe]**$^+$, recorded at different scan rates in CH_2Cl_2 referenced to $Fc^{0/+}$.

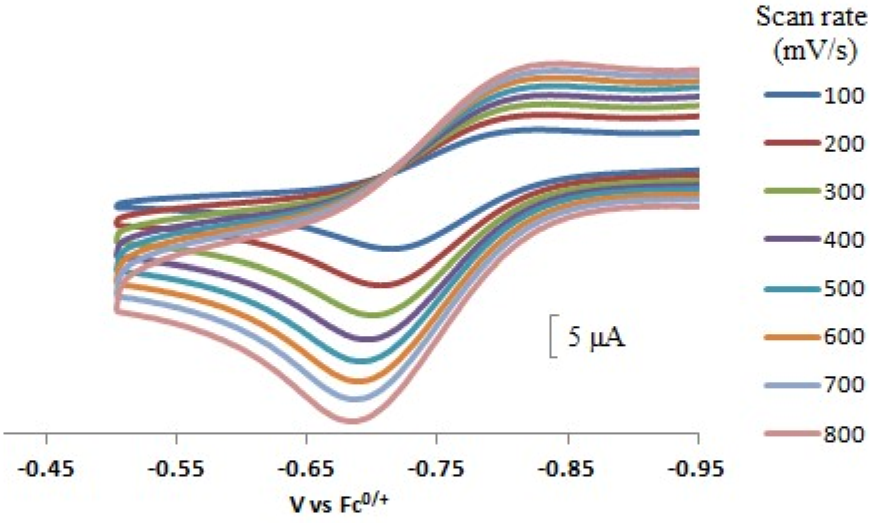

Figure S18. The reduction event, $\{Fe(NO)_2\}^{9/10}$ ($E_{1/2}$ = -0.72 V), of **[Ni-Fe]^0**, recorded at different scan rates in CH_2Cl_2 referenced to $Fc^{0/+}$.

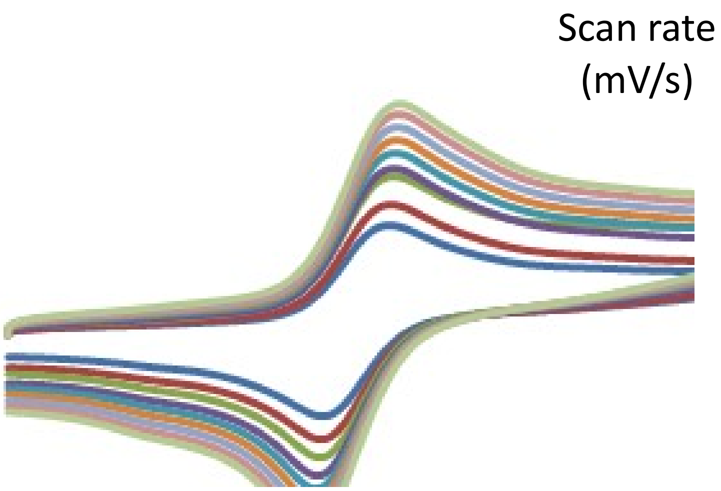

Figure S19. The reduction event, $\{Fe(NO)_2\}^{9/10}$ ($E_{1/2}$ = -0.78 V), of **[Ni$_2$-Fe]$^+$**, recorded at different scan rates in CH_3CN referenced to $Fc^{0/+}$.

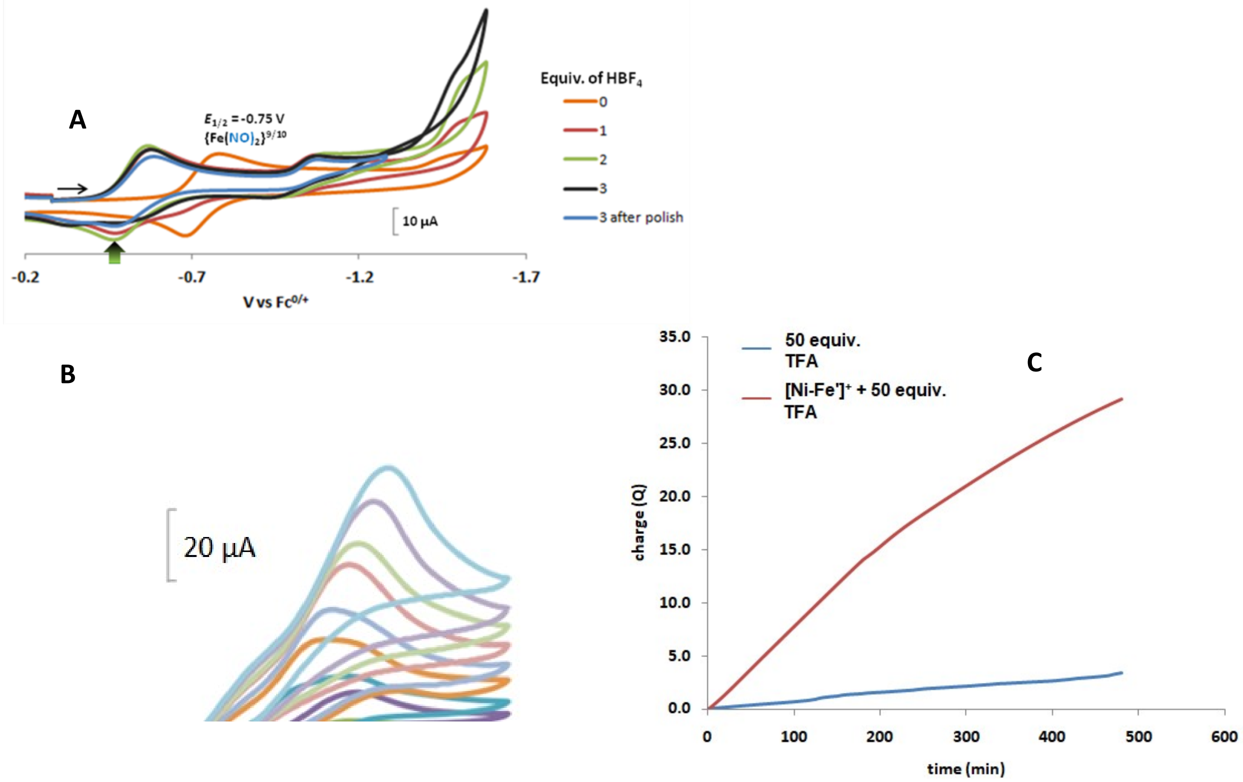

Figure S20. A) Cyclic voltammogram of **[Ni$_2$-Fe]$^+$**, in CH_3CN referenced to $Fc^{0/+}$, in presence of $HBF_4.Et_2O$. Addition of the third equivalent of $HBF_4.Et_2O$ leads to quasi-reversibility of the redox event at $E_{1/2}$ = -0.52 V, with an overall decrease in the anodic current, i_{pa}, as indicated by the green arrow. Note: the N_2S_2 ligand used in this compound is bme-dach. **B)** Cyclic voltammograms of 2.0 mM CH_2Cl_2 solutions of [**Ni-Fe]^0** in presence of 0 to 10 equivalents of $HBF_4.Et_2O$. **C)** Charge passed (in coulombs) in 480 min (8 h) for the bulk electrolysis of **[Ni-Fe']$^+$** in presence of 50 equivalents of

$HBF_4.Et_2O$ (red) and 50 equivalents of TFA only in absence of **[Ni-Fe']**$^+$ (blue), in CH_3CN at -1.73 V vs $Fc^{0/+}$. CVs of **[Ni$_2$-Fe]**$^+$ were also done in CH_3OH in presence of $HBF_4.Et_2O$ and showed similar results.

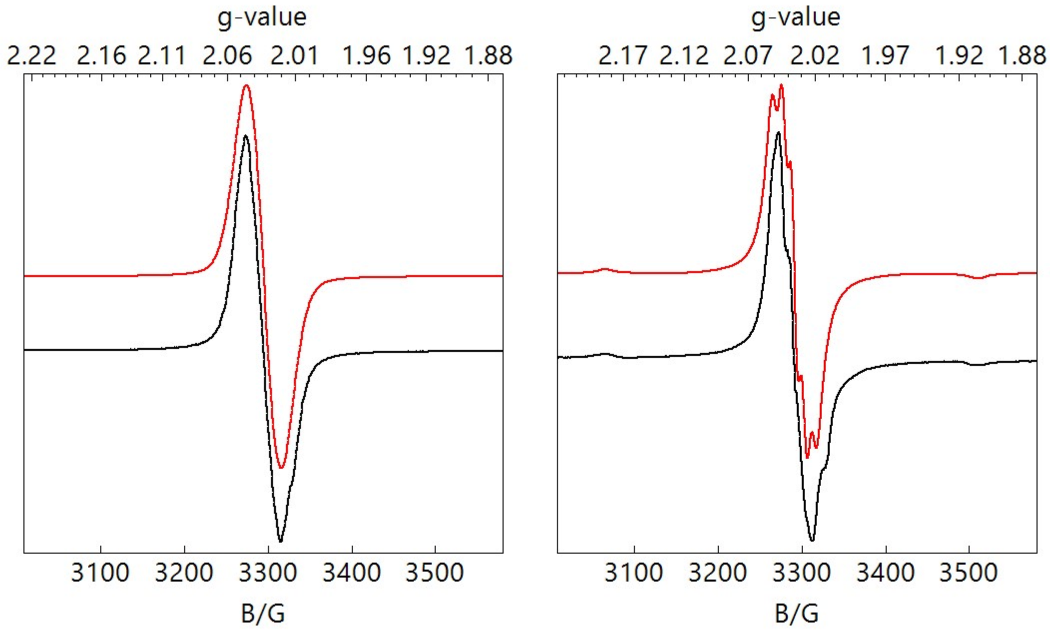

Figure S21. EPR spectrum of a CH_2Cl_2 solution of **[Ni-Fe]**$^+$ at 295 K (left) and 77 K (right). The black trace is the experimental spectrum and the red trace is the simulation (*Spin Count*). The simulated isotropic spectrum shows g = 2.033 at 295 K (left) while two species are needed to simulate the spectra at 77 K (right): species I (major) g = 2.035, $A(^{14}N)$ = 32.74 MHz and species II (minor), g_{xyz} = 2.183, 2.012, 1.908 with no observable hyperfine coupling.

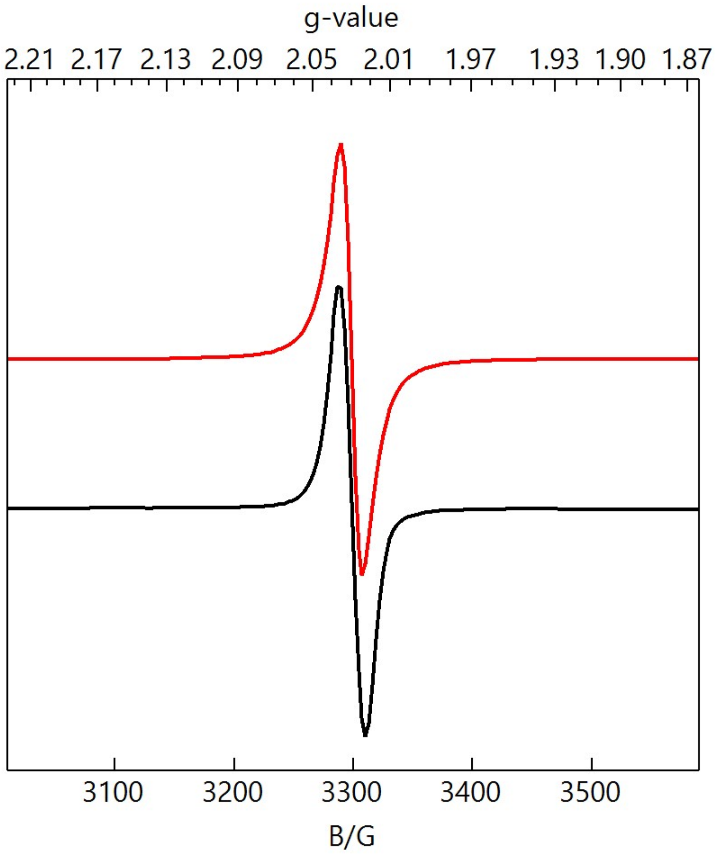

Figure S22. Frozen solution EPR spectrum of a CH_2Cl_2 solution of **[Ni$_2$-Fe]**$^+$ at 77 K. The black trace is the experimental spectrum and the red trace is the simulation (*Spin Count*). The simulated isotropic spectrum shows a *g* value of 2.029 and does not show any hyperfine coupling. Note: the N_2S_2 ligand used in this compound is bme-dach.

IR Spectrum:

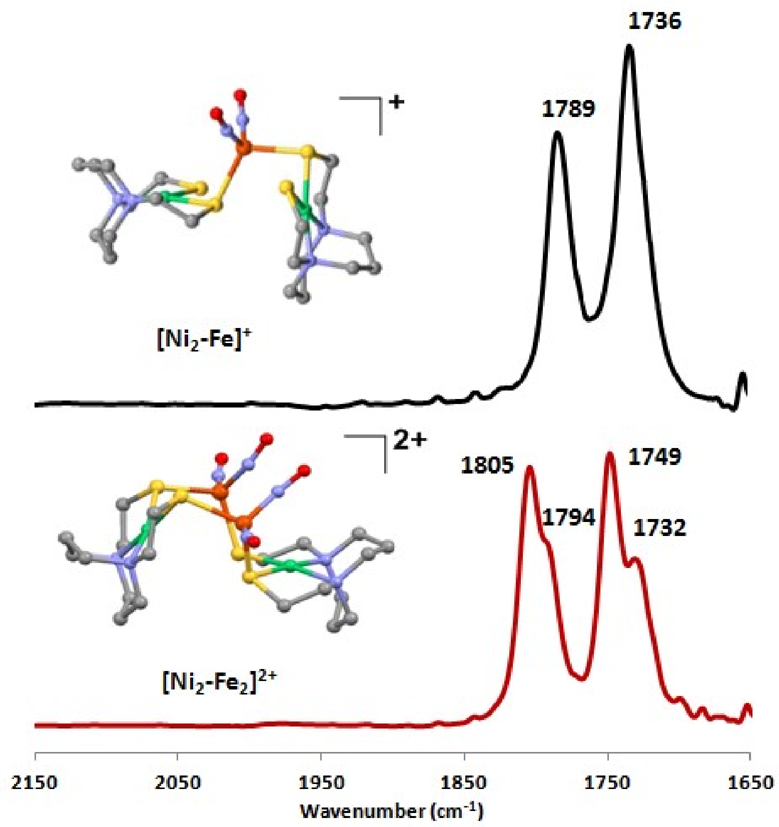

Figure S23: Stacked IR plots of **[Ni$_2$-Fe]$^+$**and **[Ni$_2$-Fe$_2$]$^{2+}$** in CH_2Cl_2 in black and red, respectively. Note: the N_2S_2 ligand used in this compound is bme-daco.

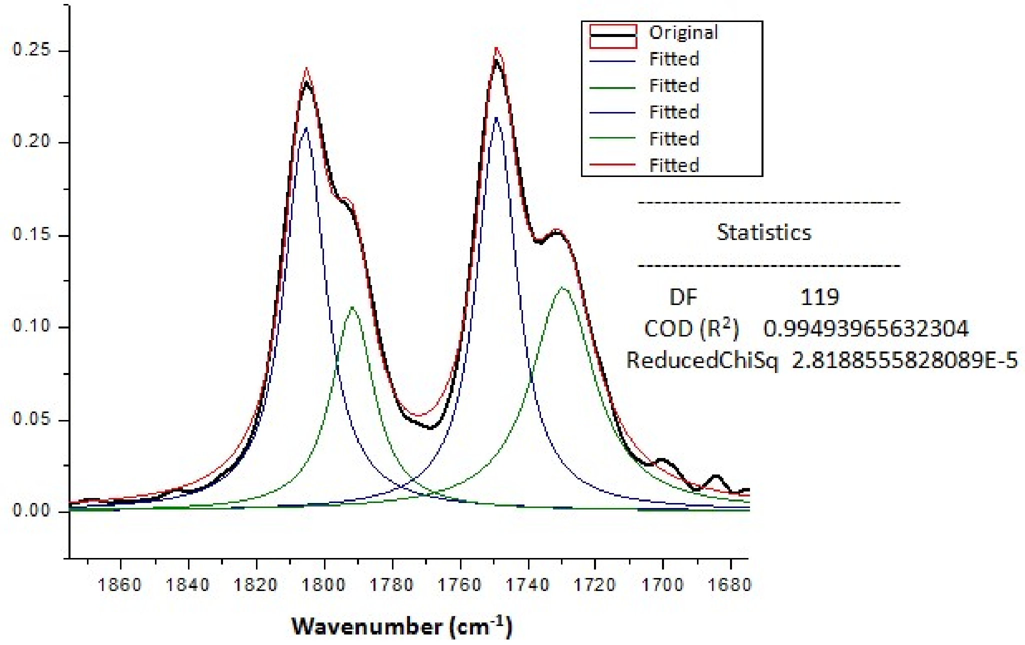

Figure S24: Deconvoluted IR spectra of **[Ni$_2$-Fe$_2$]$^{2+}$** or **[Ni-Fe]$^+$** in CH_2Cl_2 solution using Lorentzian curve fitting. Fitting parameters are shown on the right. OriginPro8 software was used for fitting.

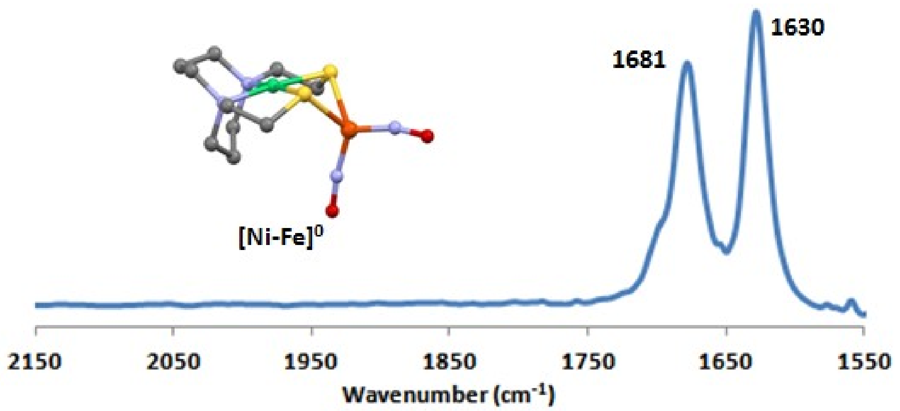

Figure S25: IR plot of **[Ni-Fe]⁰** in THF. Note: the N_2S_2 ligand used in this compound is bme-daco.

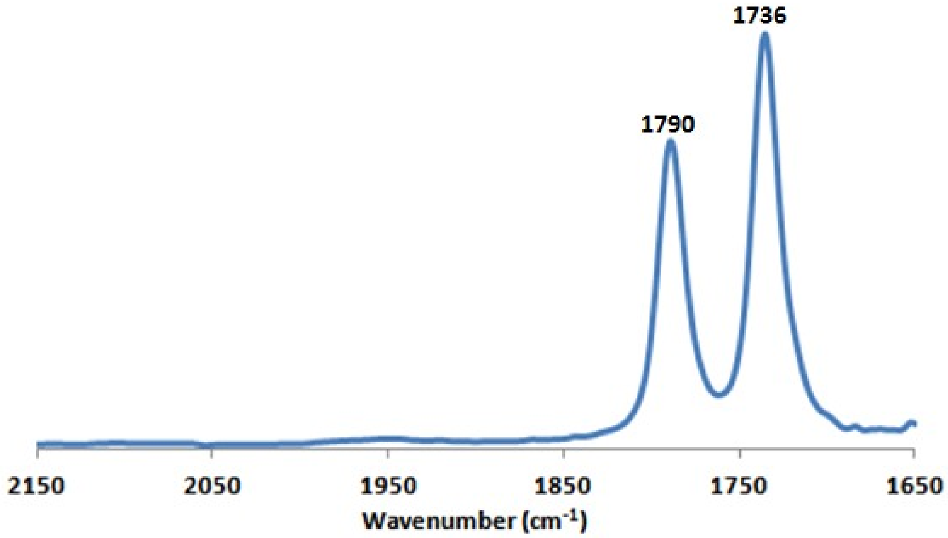

Figure S26. IR spectrum of **[Ni₂-Fe]⁺** in CH_2Cl_2. Note: the N_2S_2 ligand used in this compound is bme-dach.

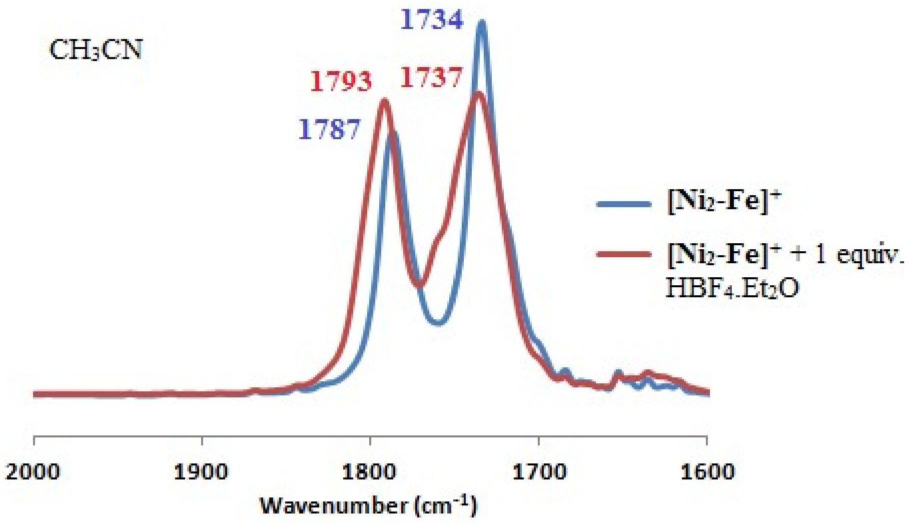

Figure S27. IR spectrum of **[Ni$_2$-Fe]$^+$** in CH_3CN (blue) and in presence of 1 equivalent of $HBF_4.Et_2O$. Note: the N_2S_2 ligand used in this compound is bme-dach.

Positive-ion ESI Mass Spectrum:

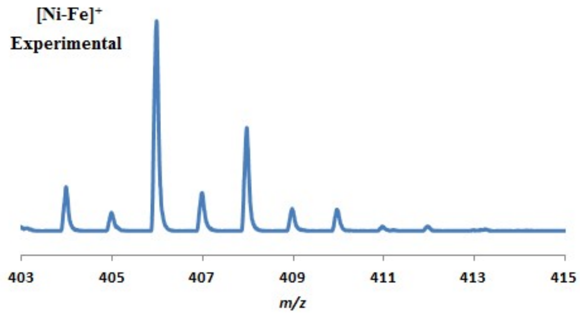

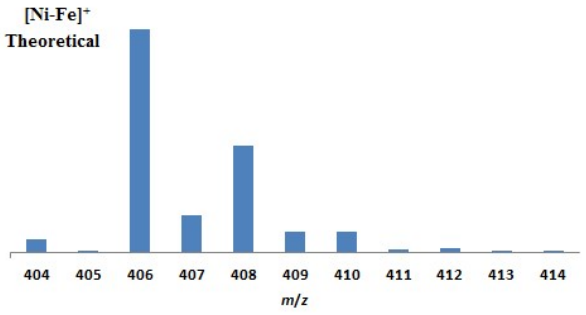

Figure S28. Positive-ion ESI mass spectrum of **[Ni-Fe]$^+$**in CH_2Cl_2.

Note: The monoisotopic mass of the crystalline dimer, **[Ni$_2$-Fe$_2$]$^{2+}$** is 811.95; while *m/z* = (811.95 / 2) = 405.97. The isotopic distribution, as shown above, matches the calculated bundle for the monomer, **[Ni-Fe]$^+$**. The difference between two consecutive isotopic mass units is ~1 rather than 0.5. This indicates the predominance of the monomer, **[Ni-Fe]$^+$**, in solution while the 0.5 value would have been consistent with the dimeric dication.

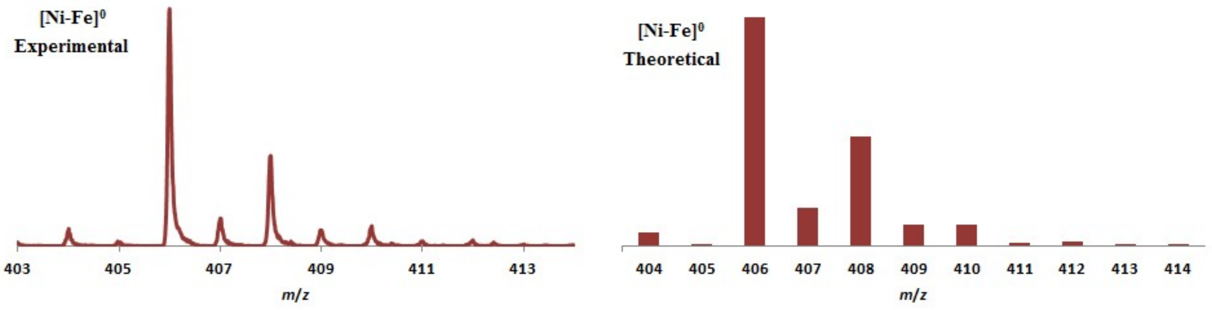

Figure S29. Positive-ion ESI mass spectrum of **[Ni-Fe]^0** in CH_2Cl_2.

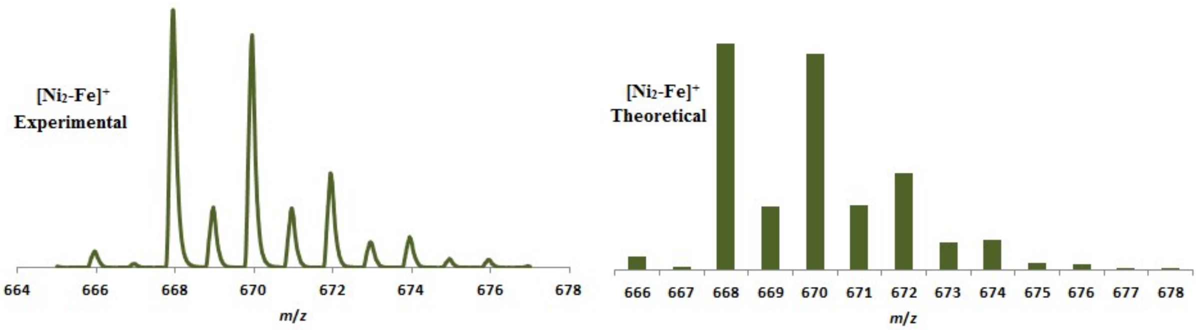

Figure S30. Positive-ion ESI mass spectrum of **[Ni-Fe]^0** in CH_2Cl_2.

Crystal Structures:

Table S6. Crystal data and structure refinement for **[Ni-Fe]^0**

Identification code	feni	
Empirical formula	C10.50 H21 Cl Fe N4 Ni O2 S2	
Formula weight	449.44	
Temperature	110(2) K	
Wavelength	0.71073 Å	
Crystal system	Monoclinic	
Space group	C 2/c	
Unit cell dimensions	a = 12.318(8) Å	□= 90°.
	b = 11.116(7) Å	□= 90.062(7)°.
	c = 24.241(15) Å	□ = 90°.
Volume	3319(4) Å^3	
Z	8	
Density (calculated)	1.799 Mg/m^3	

Absorption coefficient	2.429 mm^{-1}
F(000)	1848
Crystal size	0.400 x 0.200 x 0.050 mm^3
Theta range for data collection	2.468 to 29.638°.
Index ranges	-16<=h<=16, -15<=k<=15, -33<=l<=33
Reflections collected	20288
Independent reflections	4376 [R(int) = 0.0487]
Completeness to theta = 25.242°	99.9 %
Absorption correction	Semi-empirical from equivalents
Max. and min. transmission	0.7459 and 0.6617
Refinement method	Full-matrix least-squares on F^2
Data / restraints / parameters	4376 / 0 / 195
Goodness-of-fit on F^2	1.085
Final R indices [I>2sigma(I)]	R1 = 0.0314, wR2 = 0.0671
R indices (all data)	R1 = 0.0437, wR2 = 0.0708
Extinction coefficient	n/a
Largest diff. peak and hole	0.809 and -0.733 e.$Å^{-3}$

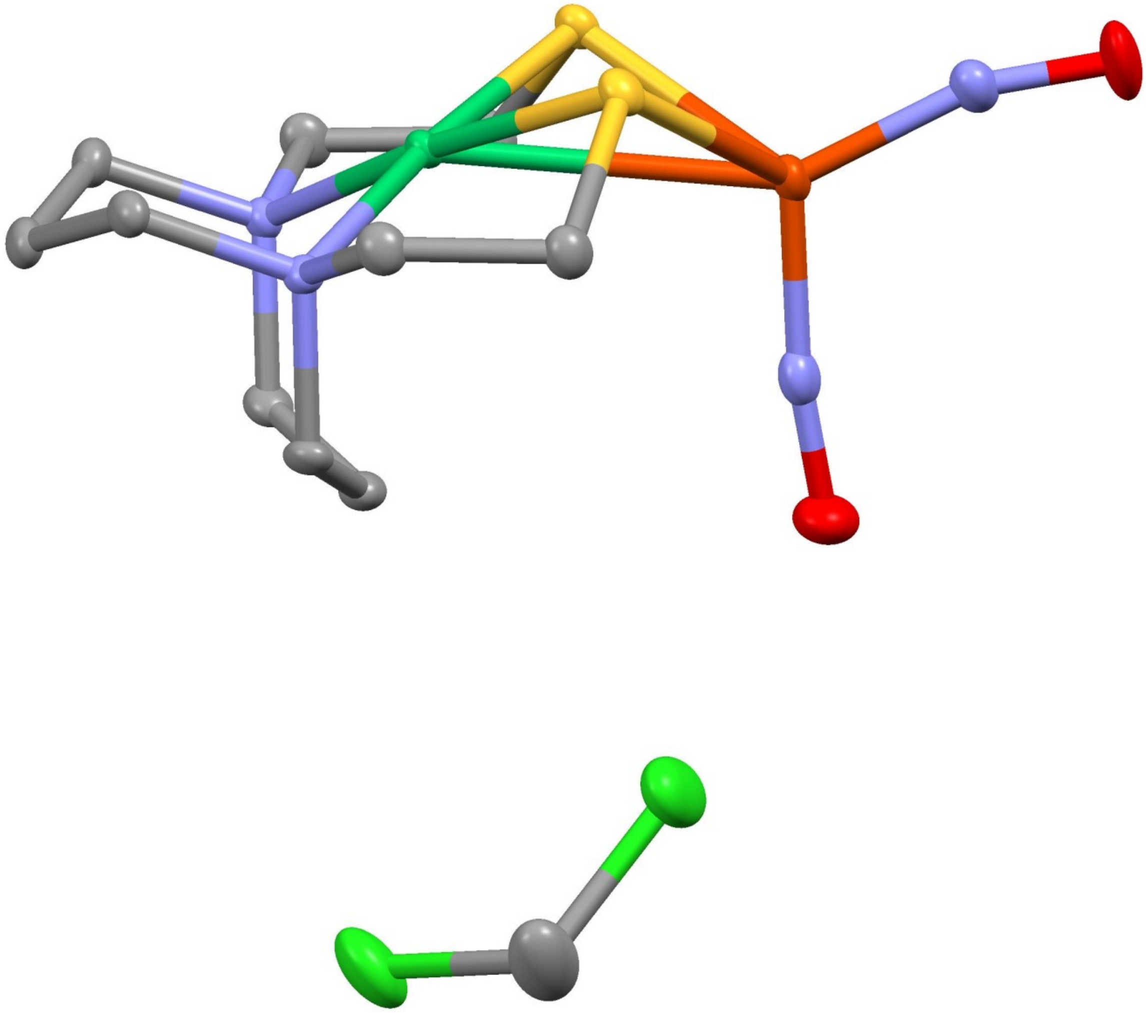

Figure S313. Thermal ellipsoid plot at 50% probability for **[Ni-Fe]^{0}**.

Table S7. Crystal data and structure refinement for **[Ni$_2$-Fe$_2$]$^{2+}$**

Identification code	temp	
Empirical formula	C22 H43.73 B1.77 Cl4 F7.08 Fe2 N8 Ni2 O4.36 S4	
Formula weight	1143.02	
Temperature	110(2) K	
Wavelength	0.71073 Å	
Crystal system	Triclinic	
Space group	P-1	
Unit cell dimensions	a = 11.6249(9) Å	α= 99.860(2)°.
	b = 14.1066(11) Å	β= 104.520(3)°.

	c = 14.2931(11) Å	γ = 105.715(2)°.
Volume	2111.1(3) $Å^3$	
Z	2	
Density (calculated)	1.798 Mg/m^3	
Absorption coefficient	2.076 mm^{-1}	
F(000)	1158	
Crystal size	0.708 x 0.499 x 0.146 mm^3	
Theta range for data collection	1.523 to 38.680°.	
Index ranges	-20<=h<=20, -24<=k<=24, -25<=l<=24	
Reflections collected	120215	
Independent reflections	23189 [R(int) = 0.0448]	
Completeness to theta = 25.242°	99.9 %	
Absorption correction	Semi-empirical from equivalents	
Max. and min. transmission	0.8685 and 0.3427	
Refinement method	Full-matrix least-squares on F^2	
Data / restraints / parameters	23189 / 238 / 597	
Goodness-of-fit on F^2	1.025	
Final R indices [I>2sigma(I)]	R1 = 0.0410, wR2 = 0.1102	
R indices (all data)	R1 = 0.0651, wR2 = 0.1255	
Extinction coefficient	n/a	
Largest diff. peak and hole	1.504 and -0.973 e.$Å^{-3}$	

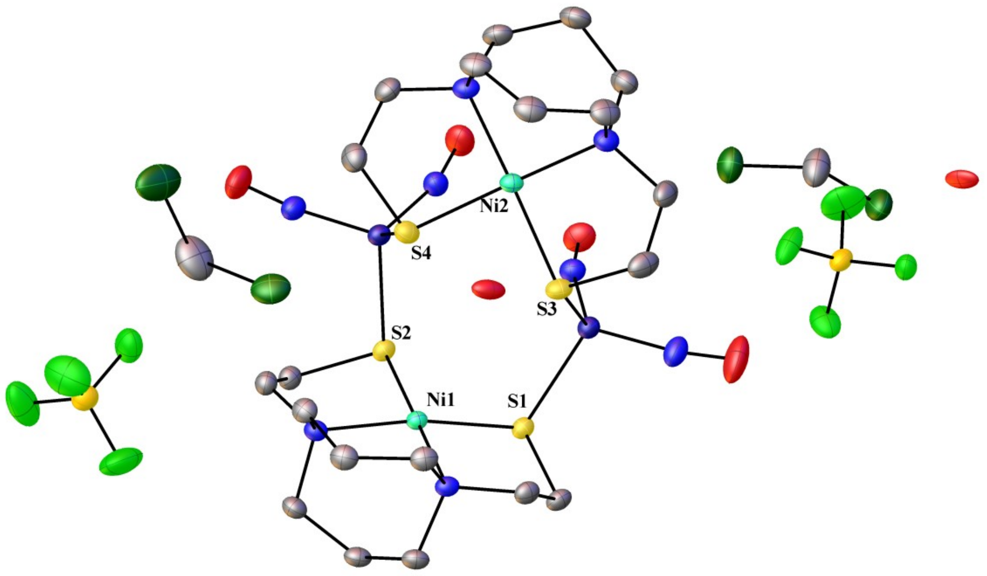

Figure S324. Thermal ellipsoid plot at 50% probability for $\mathbf{[Ni_2\text{-}Fe_2]^{2+}}$.

Table S8. Crystal data and structure refinement forfor$\mathbf{[Ni_2\text{-}Fe]^{+}}$

Identification code	nidacoox	
Empirical formula	C20 H40 B F4 Fe N6 Ni2 O2 S4	
Formula weight	784.90	
Temperature	150 K	
Wavelength	0.71073 Å	
Crystal system	Triclinic	
Space group	P-1	
Unit cell dimensions	a = 8.5324(11) Å	□= 88.197(2)°.
	b = 13.8268(18) Å	□= 72.322(2)°.
	c = 13.8581(18) Å	□ = 80.263(2)°.
Volume	1534.9(3) $Å^3$	
Z	2	
Density (calculated)	1.698 Mg/m^3	
Absorption coefficient	2.008 mm^{-1}	

F(000)	810
Crystal size	0.471 x 0.067 x 0.014 mm^3
Theta range for data collection	1.495 to 27.552°.
Index ranges	-11<=h<=11, -17<=k<=17, -17<=l<=18
Reflections collected	28965
Independent reflections	6997 [R(int) = 0.0310]
Completeness to theta = 25.242°	99.8 %
Absorption correction	Semi-empirical from equivalents
Max. and min. transmission	0.7456 and 0.6362
Refinement method	Full-matrix least-squares on F^2
Data / restraints / parameters	6997 / 503 / 445
Goodness-of-fit on F^2	1.038
Final R indices [I>2sigma(I)]	R1 = 0.0359, wR2 = 0.0981
R indices (all data)	R1 = 0.0451, wR2 = 0.1050
Extinction coefficient	n/a
Largest diff. peak and hole	2.025 and -0.449 e.$Å^{-3}$

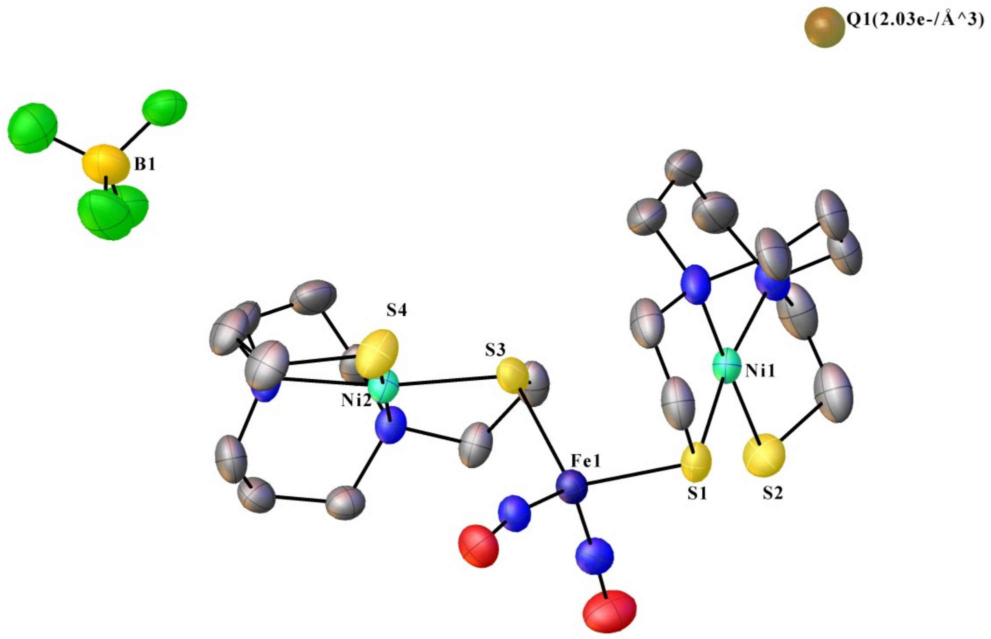

Figure S33. Thermal ellipsoid at 50 % probability for **$[Ni_2\text{-}Fe]^+$.**

Methodology for computational study

The functional TPSS[18](unless specified otherwise) was used in Gaussian 09 Revision D1[19] to execute all the calculations to keep consistence with the previous report.[17, 20] Triple-ζ 6-311++G(d,p) basis set was used for all non-metal atoms[21-23] and Wachters-Hay basis set with diffuses functions and polarization functions was applied, under the designation 6-311++G(d,p) were used for transition metals Fe and Ni.[24-26] The crystal structures of **$[Ni\text{-}Fe]^0$**, **$[Ni_2\text{-}Fe_2]^{2+}$**, **$[Ni_2\text{-}Fe]^+$**were imported as references. All the stationary points were optimized in gas phase and verified by frequency calculations with appropriate numbers of imaginary vibrations. Thermal corrections and solvation corrections (with SMD model,[27] in dichloromethane) were added to calculate Gibbs free energy. NBO analysis (version 3.1)[28] incorporated in Gaussian was applied to certain species.

Table S9. The protonation preference on **$[Ni_2\text{-}Fe]^+$** and **$[Ni_2\text{-}Fe]^0$**

Protonation Site[a]	ΔpK_a vs. $HOEt_2^+$ (*i.e.* HBF_4-OEt_2)[b]	
	**$[Ni_2\text{-}Fe]^+$**	**$[Ni_2\text{-}Fe]^0$**
**$[Ni_2\text{-}FeH]^{2+/+}$**	Not converged	16.0
**$[Ni_2\text{-}Fe\text{-}SH]^{2+/+}$**	5.7	18.5
**$[Ni_2\text{-}Fe\text{-}SHS\text{-}1]^{2+/+}$**	9.1	19.2
**$[Ni_2\text{-}Fe\text{-}SHS\text{-}2]^{2+/+}$**	9.3	20.4
**$[Ni_2\text{-}Fe\text{-}SHS\text{-}3]^{2+/+}$**	Optimized to **$[Ni_2\text{-}Fe\text{-}SHS\text{-}1]^{2+}$**	18.6

a. See Figure 8 for geometries
b. $\Delta pK_a = pK_a$(protonated species) - $pK_a(HOEt_2^+)$

Coordinates

$Ni(N_2S_2)$

0 1

Ni	0.00010600	-0.59004600	-0.20190000
S	1.52891000	-2.14376100	-0.23442500
S	-1.52837800	-2.14402600	-0.23280900
N	1.42853600	0.84133600	-0.06439300
N	-1.42855400	0.84091300	-0.06508300
C	2.90350800	-1.10596600	0.41877000
H	2.85501300	-1.03136400	1.51332200
H	3.85655900	-1.58170000	0.15786700
C	2.81163100	0.25118800	-0.24598300
H	3.55110400	0.97948000	0.13198000
H	2.95784500	0.12456000	-1.32316600
C	1.28090100	1.87928300	-1.13911400
H	1.31678500	1.33938300	-2.09164600
H	2.15159300	2.55584600	-1.09342400
C	-0.00003200	2.70344900	-1.05117500
H	-0.00032600	3.32397100	-0.14614000
H	0.00005300	3.40661200	-1.89442500
C	-1.28068800	1.87892500	-1.13972400
H	-2.15160700	2.55522400	-1.09444300
H	-1.31596400	1.33899500	-2.09225800
C	1.31537800	1.46710000	1.31025500
H	1.47386000	2.55236800	1.22840100
H	2.13402900	1.06846700	1.91767600
C	-0.00054300	1.13050900	2.02774600
H	-0.00083400	1.65966900	2.99371300
H	-0.00046900	0.05298000	2.23977200
C	-1.31621200	1.46672600	1.30961000
H	-2.13505200	1.06786300	1.91663300
H	-1.47498200	2.55193600	1.22765000
C	-2.81149100	0.25068600	-0.24727900
H	-2.95699400	0.12342500	-1.32448500
H	-3.55125900	0.97919100	0.12973400
C	-2.90376400	-1.10594100	0.41835500

H	-3.85648200	-1.58199500	0.15683300
H	-2.85633500	-1.03053200	1.51289900

$[Ni_2\text{-}Fe_2]^{2+}$

2 3

Ni	-2.80164800	0.10582200	0.07772600
Ni	2.45987400	-0.06243300	-0.69833100
Fe	0.09014600	2.30910000	1.08622100
Fe	-0.08423900	-2.06527600	1.12693000
S	-2.18538500	1.87714500	1.24048500
S	-2.14292900	-1.16500300	1.75929200
S	1.06526200	1.62538700	-0.92774900
S	0.75940000	-1.43637400	-0.97080300
O	0.43254600	5.10612200	1.26124500
O	1.30036900	1.42493600	3.46837700
O	-0.28896800	-4.87992800	1.12433200
O	1.72128400	-1.61858900	3.23986900
N	0.23382500	3.96926400	1.06958900
N	0.77418700	1.60221100	2.43867600
N	-0.29100300	-3.71321300	1.01508700
N	0.96623000	-1.61656100	2.34461900
N	-3.49210300	1.29818700	-1.38760400
N	-3.74859300	-1.46483600	-0.76474700
N	4.00955200	1.21149300	-0.59892900
N	3.73811400	-1.61768700	-0.71350800
C	-3.00634800	3.16447000	0.18809300
H	-4.03073900	3.27374100	0.55860100
H	-2.48844200	4.11672200	0.32490500
C	-2.94246900	2.70398300	-1.25101600
H	-3.50667400	3.36632600	-1.92415500
H	-1.90010900	2.67126500	-1.58297300
C	-3.02303500	0.79749200	-2.73396600
H	-1.93000500	0.73953600	-2.67824200
H	-3.28912800	1.55580600	-3.48510800
C	-3.60961900	-0.55013500	-3.14359300
H	-4.69049600	-0.47485900	-3.30878900
H	-3.18211100	-0.81032100	-4.11976600
C	-3.28209900	-1.68919400	-2.18477800
H	-3.74252800	-2.61960600	-2.54870200
H	-2.19807800	-1.83926900	-2.12987100
C	-5.01297400	1.33979500	-1.31700300
H	-5.41485100	1.26065400	-2.33356300
H	-5.30064800	2.32292100	-0.93447600
C	-5.59389600	0.27077800	-0.38768500
H	-6.68822400	0.36491600	-0.40476600
H	-5.27566000	0.48633200	0.64261600
C	-5.24765000	-1.18472000	-0.71858100
H	-5.67464900	-1.82085100	0.06177900
H	-5.68970800	-1.50218700	-1.67070800
C	-3.46426800	-2.75622500	-0.02367900
H	-2.49917200	-3.12614100	-0.38330600
H	-4.23791700	-3.49182100	-0.28629700

C	-3.38099600	-2.51271700	1.46657200
H	-3.05871900	-3.40856000	2.00473300
H	-4.32612100	-2.17086900	1.90074600
C	2.34120200	2.90472100	-1.34628200
H	2.56533700	2.80206800	-2.41282700
H	1.92289300	3.89913600	-1.17110400
C	3.54257200	2.64744200	-0.46253800
H	4.38361000	3.31418100	-0.70116300
H	3.26413600	2.80061100	0.58476600
C	4.82571700	1.06679900	-1.87857800
H	5.89061000	1.08035100	-1.61887200
H	4.63388700	1.95134600	-2.49241600
C	4.45239900	-0.17385700	-2.69555900
H	5.10335600	-0.20079500	-3.58033000
H	3.42286200	-0.05647300	-3.06380600
C	4.56982400	-1.52790900	-1.98834800
H	5.61006000	-1.77294200	-1.74388600
H	4.20996000	-2.30244700	-2.67177100
C	4.86115300	0.93342800	0.62143100
H	4.19558500	1.00372000	1.48884700
H	5.60730800	1.73741700	0.70031900
C	5.56864400	-0.41672000	0.59945700
H	6.32288500	-0.45177200	-0.19499700
H	6.12713400	-0.50878500	1.53897500
C	4.62646100	-1.61174500	0.51194200
H	5.21237700	-2.54232700	0.50704800
H	3.95969300	-1.63553100	1.38025500
C	3.00504500	-2.94404700	-0.68795900
H	2.71656100	-3.13519200	0.35029600
H	3.69644300	-3.73593700	-1.00942900
C	1.76195500	-2.88481900	-1.54928200
H	1.16648100	-3.79673600	-1.45354300
H	1.98434800	-2.71969400	-2.60836000

[Ni-Fe]^0

0 1

Ni	-0.54428200	-0.00076100	-0.59805400
Fe	2.23924000	-0.00142500	0.09869500
S	0.95442600	-1.49011700	-1.15832200
S	0.95604900	1.48579500	-1.16153800
O	5.00562300	-0.00062300	-0.43529300
O	1.88513200	0.00734700	2.91824200
N	3.81750200	-0.00241400	-0.39807000
N	1.98871700	0.00286400	1.71891400
N	-1.81661000	-1.42924300	0.02126000
N	-1.81467000	1.43043200	0.01903800
C	0.24547800	-2.81921500	-0.06968400
H	0.66996200	-3.78249300	-0.37345900
H	0.52404900	-2.62281500	0.96971700
C	-1.25444300	-2.79712000	-0.30225900

H	-1.45343600	-2.98141200	-1.36332300
H	-1.79445300	-3.55229300	0.29229000
C	-2.03852400	-1.31153700	1.51768700
H	-1.48788700	-2.12842300	1.99418800
H	-3.10439800	-1.47470600	1.73150900
C	-1.51752000	0.00189900	2.11164800
H	-0.42543600	0.00094100	2.02876300
H	-1.75608900	0.00294900	3.18542700
C	-2.03642800	1.31528900	1.51568700
H	-3.10199400	1.48054900	1.72946200
H	-1.48432600	2.13196800	1.99083500
C	-3.11383500	-1.28890600	-0.72366400
H	-3.75563500	-2.15210400	-0.47852500
H	-2.86490300	-1.33793700	-1.78948200
C	-3.87277900	0.00160500	-0.41533800
H	-4.78250500	0.00176400	-1.02999300
H	-4.21835500	0.00257800	0.62558500
C	-3.11216800	1.29068800	-0.72551300
H	-2.86328600	1.33790700	-1.79142700
H	-3.75284000	2.15505700	-0.48153500
C	-1.25078700	2.79706900	-0.30671600
H	-1.78952800	3.55386700	0.28691700
H	-1.44998100	2.98008500	-1.36796500
C	0.24927600	2.81760700	-0.07481000
H	0.52804700	2.62264100	0.96481200
H	0.67483700	3.77984300	-0.38038200

$[Ni_2\text{-}Fe]^+$

1 2

Ni	-2.70675600	-0.17615700	-0.09674500
Ni	2.44001600	0.42253000	-0.24064500
Fe	-0.29859800	-1.20922100	1.48822500
S	-2.85165800	-2.16517400	-0.97411500
S	-2.54556800	-0.91382600	1.94810100
S	0.41762700	-0.31382300	-0.57788600
S	1.76392500	2.37211700	0.43269000
O	0.09409400	-4.00569900	1.61304500
O	1.15520600	-0.03763500	3.61213700
N	-3.06557100	0.66427600	-1.82384300
N	-2.78957500	1.69249800	0.53043700
N	-0.13584200	-2.86534400	1.44429800
N	0.53541500	-0.40258600	2.68222600
C	-3.36230100	-1.63825300	-2.67898800
H	-2.98039100	-2.37435300	-3.39408000
H	-4.45484400	-1.63235000	-2.75805300
C	-2.76270200	-0.27237300	-2.96557100
H	-1.67269600	-0.35052200	-3.01401000
H	-3.13825500	0.16885100	-3.90332500
C	-4.53579700	1.01165200	-1.80431000
H	-5.07474500	0.10727600	-1.50812300

H	-4.84234100	1.28664800	-2.82537000
C	-4.84471000	2.17513400	-0.85519100
H	-5.93414100	2.25849400	-0.76442200
H	-4.50696700	3.11796500	-1.30183300
C	-4.26106800	2.03179400	0.55530900
H	-4.39332300	2.97910600	1.10091200
H	-4.76829100	1.23559700	1.10852300
C	-2.21984400	1.90564800	-1.89457300
H	-2.64713300	2.60924600	-2.62279400
H	-1.23545400	1.59086500	-2.24735400
C	-2.06874000	2.53510000	-0.48512600
H	-1.01160600	2.54846300	-0.20786400
H	-2.44197700	3.56776100	-0.44814600
C	-2.18260800	1.84008900	1.89907200
H	-2.42104800	2.83685500	2.30211900
H	-1.09811500	1.76021500	1.77479600
C	-2.67938500	0.73288700	2.81440000
H	-3.73071700	0.85529900	3.09438700
H	-2.08386200	0.68835900	3.73050700
C	0.89713300	-1.80792200	-1.58201000
H	0.09214000	-2.54262200	-1.48733900
H	0.96762800	-1.49539400	-2.62943900
C	2.21921400	-2.34200700	-1.05244100
H	2.61581000	-3.14745900	-1.68985100
H	2.07523600	-2.73085400	-0.04138100
N	3.23205900	-1.23014900	-0.95466900
C	3.74885800	-0.86726400	-2.32628900
H	4.01307900	-1.79458300	-2.85851700
H	2.92737200	-0.38181300	-2.86210100
C	4.97937000	0.04501300	-2.26591100
H	5.19520500	0.39090400	-3.28375900
H	5.85542300	-0.53867100	-1.95763800
C	4.83430900	1.27834300	-1.36499100
H	5.81761200	1.76045900	-1.25069200
H	4.13661300	2.00279600	-1.79495600
C	4.36611800	-1.58619000	-0.03525900
H	5.10133400	-2.21302700	-0.55907800
H	3.94055400	-2.17651200	0.77927300
C	5.00700300	-0.29434200	0.54484500
H	4.87307900	-0.27917000	1.62857400
H	6.08432000	-0.23831100	0.33534800
N	4.30944900	0.92306700	0.00445100
C	4.44184000	2.09714500	0.94728100
H	5.48007800	2.46567000	0.91847500
H	4.22103800	1.71806600	1.94929200
C	3.43147400	3.17069900	0.57987000
H	3.69202000	3.67879900	-0.35492000
H	3.38551900	3.92394500	1.37278200

[Ni_2-Fe]

0 1

Ni	-2.73712600	0.34319600	-0.03039700
Ni	2.16914300	-0.46799400	0.52454300
Fe	0.39581300	1.05811300	-1.53900000
S	-3.08592600	2.30858200	0.87675600
S	-1.88878200	1.13399300	-1.89320300
S	0.18579800	0.36957500	0.72892200
S	1.53110400	-2.56092000	0.41796200
O	1.42348000	3.60497800	-2.21883800
O	1.64261200	-0.85036700	-3.22470800
N	-3.43161100	-0.56878500	1.57484200
N	-2.79200000	-1.49697000	-0.75986600
N	0.92312700	2.59191000	-1.82056600
N	1.08840800	-0.12666000	-2.44430700
C	-3.80231400	1.70753400	2.48181700
H	-3.49622600	2.39124600	3.28166200
H	-4.89860400	1.71983200	2.43869900
C	-3.27127700	0.31264100	2.77920900
H	-2.19557500	0.36591500	2.97331000
H	-3.77568800	-0.15535100	3.64411200
C	-4.88105800	-0.84858800	1.30368100
H	-5.32956200	0.09403300	0.97662400
H	-5.36755500	-1.16882300	2.24135200
C	-5.06842900	-1.94167500	0.24393500
H	-6.12985400	-1.97091000	-0.03240200
H	-4.84274100	-2.92185100	0.68278000
C	-4.24798800	-1.75505600	-1.04011400
H	-4.33383200	-2.66911600	-1.65251000
H	-4.62272000	-0.90845500	-1.62301000
C	-2.64257300	-1.83589700	1.71264200
H	-3.18895500	-2.56065800	2.33599400
H	-1.71410900	-1.56866900	2.22243100
C	-2.28875700	-2.41286000	0.31525900
H	-1.20167100	-2.46531300	0.21206400
H	-2.69255200	-3.42688500	0.17175600
C	-1.97727100	-1.62164900	-2.01730800
H	-2.23753600	-2.56199000	-2.53237100
H	-0.92636700	-1.65971000	-1.71322100
C	-2.19788200	-0.40295300	-2.89847000
H	-3.21818900	-0.35030400	-3.29602400
H	-1.49579300	-0.40389300	-3.73729300
C	0.70556600	1.81744200	1.78142000
H	-0.08221300	2.57587300	1.71400600
H	0.77194800	1.46165400	2.81626400
C	2.03317000	2.35459500	1.26900700
H	2.45901300	3.11191600	1.94693000

H	1.87241300	2.80363200	0.28857600
N	3.02775400	1.23476200	1.08104900
C	3.71326700	0.91947800	2.38187100
H	4.03831500	1.86510300	2.84924900
H	2.96820000	0.45033700	3.03269900
C	4.94310300	0.01778300	2.20369400
H	5.29725000	-0.27334900	3.20055300
H	5.75654600	0.60082700	1.75327300
C	4.71224100	-1.25807800	1.38506000
H	5.68597700	-1.73007500	1.16719100
H	4.08550900	-1.96881800	1.93180000
C	4.02351000	1.54293600	0.00218400
H	4.82065400	2.19934100	0.38508600
H	3.47730900	2.07701300	-0.77732300
C	4.59478400	0.22467100	-0.59846800
H	4.29758400	0.15465200	-1.64727400
H	5.69445100	0.19201800	-0.54782800
N	4.00747700	-0.96969200	0.09303200
C	3.98817200	-2.17864200	-0.80216400
H	5.02556900	-2.51178800	-0.98499700
H	3.53713900	-1.85565300	-1.74483600
C	3.13571500	-3.28173100	-0.18657600
H	3.65281000	-3.77945500	0.64310900
H	2.92362800	-4.03711100	-0.95048100

[Ni_2-FeH]$^+$

1 1

Ni	-2.75541300	-0.11318000	-0.20928100
Ni	2.32959200	0.54792800	-0.03447700
Fe	0.10814300	-2.15995900	0.94860000
S	-2.94901600	-1.33180100	-2.00129000
S	-2.23382500	-1.81441100	1.05967300
S	0.33946800	-0.22431300	-0.47936500
S	1.61182800	1.84479100	1.55288700
O	0.79404900	-4.44839700	-0.53362500
O	1.86688300	-1.62663200	3.07354100
N	-3.31636600	1.48943000	-1.16983600
N	-2.81763800	1.10291500	1.33801900
N	0.45005900	-3.44778400	-0.03179700
N	1.11853500	-1.71613700	2.17961600
C	-3.63999200	-0.01450200	-3.11155200
H	-3.31260600	-0.21740700	-4.13657400
H	-4.73480300	-0.05069900	-3.09804000
C	-3.11114100	1.33464900	-2.65609900
H	-2.03079000	1.38019900	-2.82385900
H	-3.59365300	2.17856000	-3.17652100
C	-4.78025000	1.67914300	-0.85684800
H	-5.27462000	0.72540700	-1.06201100
H	-5.18926100	2.44332000	-1.53619200
C	-5.00858100	2.11999500	0.59341700

H	-6.08451800	2.06939100	0.79840300
H	-4.73053400	3.17447500	0.70840100
C	-4.28525800	1.27562400	1.64943800
H	-4.37962700	1.76612600	2.63110300
H	-4.72522500	0.27611900	1.71552300
C	-2.49184000	2.62023900	-0.61989400
H	-2.98591200	3.58175200	-0.82048100
H	-1.54219300	2.60427500	-1.15947900
C	-2.21423500	2.40498700	0.89227800
H	-1.13554900	2.33727000	1.05497000
H	-2.60048200	3.23060000	1.50631600
C	-2.08969100	0.52755400	2.52330100
H	-2.33383500	1.11194800	3.42470500
H	-1.01810600	0.62656300	2.32236400
C	-2.45348000	-0.93935700	2.68934900
H	-3.49672700	-1.08147100	2.98945000
H	-1.81397300	-1.42114100	3.43348300
C	0.77643400	-0.91037500	-2.15592700
H	0.04878500	-1.68684000	-2.40608700
H	0.65536500	-0.09282300	-2.87443100
C	2.20177300	-1.43872600	-2.12332900
H	2.54605200	-1.73716300	-3.12577500
H	2.25853800	-2.30627400	-1.46214900
N	3.13264800	-0.39766300	-1.55708600
C	3.41349700	0.67223200	-2.58628700
H	3.66223800	0.18290600	-3.54098400
H	2.48845600	1.24179000	-2.71843800
C	4.57134500	1.59131300	-2.17722600
H	4.61298400	2.42256700	-2.89097700
H	5.52212500	1.05545300	-2.28696400
C	4.46931900	2.17886400	-0.76437600
H	5.42124500	2.66845100	-0.50607000
H	3.66577300	2.91819500	-0.69986400
C	4.41505500	-1.00151400	-1.05832800
H	5.10990600	-1.16777600	-1.89335400
H	4.16400100	-1.97480500	-0.63108700
C	5.03263900	-0.09677700	0.04517200
H	5.05509400	-0.64277700	0.99081400
H	6.06167600	0.20417100	-0.19603300
N	4.17455600	1.11891900	0.26588000
C	4.34810100	1.66509200	1.66303200
H	5.34736200	2.12158500	1.75036500
H	4.28165100	0.80935800	2.34120800
C	3.23133600	2.64590100	1.97841200
H	3.34219000	3.58761200	1.43014600
H	3.23224100	2.87553400	3.04852700
H	-0.46747000	-3.15025300	1.91062500

[Ni_2-Fe-SH]$^+$

1 1

Ni	2.95676300	0.42226300	0.22612300
Ni	-2.84292000	0.25798500	-0.46932000
Fe	-0.17060000	-0.78120700	0.18727500
S	2.47031700	2.49232800	-0.21939000
S	1.34771700	0.18205000	1.67741800
S	-0.90636200	1.22220600	-0.66187300
S	-2.86340100	-0.62179400	-2.52795900
O	-1.82212100	-2.54145700	1.67820000
O	0.87060100	-2.24821500	-2.00153700
N	4.38177200	0.42891500	-1.09064800
N	3.73707400	-1.32465300	0.69841100
N	-1.18836100	-1.68833200	1.11548300
N	0.50657600	-1.53838400	-1.10804500
C	3.74691400	2.78442400	-1.53535400
H	3.32021600	3.44744600	-2.29464500
H	4.61931600	3.28616700	-1.10215000
C	4.11154400	1.45205300	-2.16676200
H	3.26067500	1.07506900	-2.74160600
H	4.99336800	1.52276700	-2.82497200
C	5.64733200	0.75815100	-0.34079600
H	5.44658800	1.66376100	0.23839600
H	6.44514500	0.97018700	-1.07015100
C	6.08819700	-0.39552000	0.56915900
H	6.89634700	-0.03154100	1.21466500
H	6.52636700	-1.19746200	-0.03799000
C	4.98869100	-0.97690400	1.46954400
H	5.36865000	-1.89067000	1.95363200
H	4.70117900	-0.26340700	2.24771500
C	4.44580700	-0.95792200	-1.66999900
H	5.43760600	-1.13600200	-2.10951600
H	3.70604500	-0.99924800	-2.47245500
C	4.08437300	-2.01357800	-0.59056700
H	3.19698400	-2.56199800	-0.91129100
H	4.89703900	-2.73622100	-0.42959200
C	2.80801500	-2.15699200	1.54057100
H	3.37348600	-2.97105900	2.02058600
H	2.06292100	-2.58652500	0.86426200
C	2.09516300	-1.28098200	2.55885000
H	2.77003500	-0.88974300	3.32749500
H	1.29143700	-1.83857100	3.04914700
C	-1.17508400	2.48984500	0.67700700
H	-0.19068600	2.79149300	1.04730000
H	-1.65374900	3.35938100	0.21529100
C	-2.00291300	1.86030400	1.77820900
H	-2.29485100	2.58634500	2.55265900
H	-1.41937200	1.05537800	2.23540700
N	-3.24324100	1.21735800	1.20190500
C	-4.28037800	2.25478200	0.85126900
H	-4.38371400	2.95008100	1.69944000
H	-3.90434500	2.81351700	-0.01062800
C	-5.64944200	1.62507700	0.55765400
H	-6.30325900	2.40533500	0.15048800

H	-6.11527300	1.30439800	1.49696600
C	-5.63890800	0.45418900	-0.43460100
H	-6.63156200	-0.02391400	-0.44069000
H	-5.41816100	0.81109500	-1.44573100
C	-3.81209000	0.20353400	2.15648400
H	-4.41873800	0.70832600	2.92102000
H	-2.96537800	-0.27401800	2.65161600
C	-4.61951800	-0.87849200	1.38831600
H	-4.13042300	-1.84485200	1.52735300
H	-5.65567700	-0.95541300	1.74641400
N	-4.60191700	-0.58278100	-0.08388800
C	-4.77723500	-1.82849300	-0.89898800
H	-5.80583100	-2.20919300	-0.79576100
H	-4.08548300	-2.57243400	-0.49255900
C	-4.45809300	-1.56780000	-2.36990800
H	-5.21081300	-0.94770300	-2.86354700
H	-4.36863700	-2.50169900	-2.93016500
H	-2.02716300	-1.67278800	-2.32349800

[Ni_2-Fe-SHS-1]$^+$

1 1

Ni	-2.54483700	0.26400500	-0.44130800
Ni	2.59377700	0.24559300	-0.40897100
Fe	-0.16543300	-1.43047300	0.03923800
S	-1.69866600	1.88728900	-1.68386800
S	-1.78299300	-1.36058400	-1.65559600
S	1.53730600	-1.25453000	-1.58037100
S	1.78991000	1.94712300	-1.52413500
O	-0.10239000	-3.98889500	1.25406400
O	-0.00641200	0.60336400	2.02586800
N	-3.27238200	1.58543700	0.81606900
N	-3.78718700	-0.93866700	0.51582300
N	-0.15122000	-2.95100500	0.66998600
N	-0.10558500	-0.15819800	1.08885200
C	-2.12669800	3.30945700	-0.56258600
H	-1.29445700	4.01832300	-0.57535600
H	-2.99709600	3.80152400	-1.00403000
C	-2.38938900	2.80089000	0.85093200
H	-1.45236000	2.48929600	1.32106500
H	-2.86076100	3.58296600	1.46764500
C	-4.65952000	1.93212600	0.33657900
H	-4.58584100	2.18799700	-0.72481900
H	-5.01113900	2.81843800	0.88800500
C	-5.64455600	0.77743300	0.54898200
H	-6.58009500	1.03016400	0.03627300
H	-5.89752100	0.69857600	1.61306500
C	-5.17272500	-0.58650600	0.03171400
H	-5.87333700	-1.36000300	0.38295200
H	-5.14945200	-0.61262600	-1.06204800
C	-3.32528200	0.90608100	2.15649500
H	-4.06661400	1.40102000	2.79874400

C	-3.61908900	-0.60797000	1.97350500
H	-2.75520100	-1.17874000	2.32056200
H	-4.50220900	-0.93235300	2.54189100
C	-3.48954900	-2.39679600	0.25760400
H	-4.36145400	-3.00371500	0.54505700
H	-2.64311800	-2.66059500	0.89505100
C	-3.09388300	-2.60466200	-1.19199900
H	-3.92828200	-2.46810000	-1.88732500
H	-2.68063200	-3.60749400	-1.33898800
C	2.69962200	-2.65814100	-1.19043200
H	2.15660200	-3.59976600	-1.31579600
H	3.50912500	-2.62338300	-1.92701300
C	3.19593900	-2.51408300	0.23772000
H	3.98773500	-3.24009800	0.47900000
H	2.35533900	-2.65887300	0.91997600
N	3.70681400	-1.11553400	0.47205400
C	5.07550300	-0.94122800	-0.14060200
H	5.69785200	-1.80510200	0.14115800
H	4.94520400	-0.94572100	-1.22701200
C	5.77032300	0.34616000	0.32597900
H	6.67676000	0.47849300	-0.27634800
H	6.11300000	0.22502100	1.36104300
C	4.92795500	1.62312200	0.20575100
H	5.43577700	2.44599300	0.73349900
H	4.79261400	1.90467600	-0.84242500
C	3.72952600	-0.78014300	1.93525400
H	4.63001600	-1.19613700	2.40897400
H	2.85667100	-1.26002000	2.38265000
C	3.62037000	0.75611300	2.12926300
H	2.68666200	0.98363400	2.64820900
H	4.45394400	1.16168000	2.71985700
N	3.55142900	1.44518500	0.79493500
C	2.83430600	2.76475500	0.90633100
H	3.45337700	3.46645600	1.48967200
H	1.90799700	2.56221000	1.45191700
C	2.51375200	3.30566700	-0.47885100
H	3.40497500	3.69177600	-0.98409700
H	1.79553200	4.12803900	-0.40170800
H	-2.33831000	1.02331800	2.60843200
H	-0.28157900	1.88795400	-1.42990600

$[Ni_2\text{-Fe-SHS-2}]^+$

1 1

Ni	2.59404300	0.34025400	0.43659600
Ni	-2.20288900	0.51806600	-0.37690400
Fe	-0.30500300	-1.81688100	0.81431100
S	1.87200900	2.00403800	1.70881800
S	1.72277700	-1.24258400	1.67064400
S	-1.14642300	2.39624100	-0.01022300

S	-0.48606700	-0.56872800	-1.18092100
O	-2.15419100	-1.29107500	2.89500000
O	-0.18245800	-4.55991900	0.11066400
N	3.34376700	1.63088000	-0.88965200
N	3.54311500	-0.95792900	-0.69298700
N	-1.37121800	-1.37464700	1.99854300
N	-0.21323700	-3.39538700	0.35240200
C	2.52494100	3.39883100	0.66617100
H	1.83650200	4.24381800	0.75527200
H	3.48395300	3.68947200	1.10342100
C	2.63967000	2.95152200	-0.78847400
H	1.63592000	2.81391900	-1.20149900
H	3.16991600	3.70845500	-1.38839800
C	4.80915100	1.77262000	-0.57899700
H	4.89509600	2.00295200	0.48790300
H	5.21695400	2.62084300	-1.15190800
C	5.59776500	0.50345400	-0.92328400
H	6.61278700	0.61456400	-0.52381900
H	5.71098300	0.42388300	-2.01117500
C	5.01077400	-0.80146100	-0.37069200
H	5.55680000	-1.65115200	-0.80944200
H	5.11213700	-0.85332600	0.71769400
C	3.12791700	0.98874700	-2.22751300
H	3.81784400	1.40939400	-2.97293100
H	2.10625600	1.23015700	-2.53058900
C	3.26251000	-0.55653700	-2.11391100
H	2.30627400	-1.01088100	-2.38022700
H	4.03874700	-0.95499200	-2.78229500
C	3.09407600	-2.37358500	-0.42196300
H	3.82776900	-3.07795700	-0.84347800
H	2.13381300	-2.50705200	-0.92874100
C	2.88643800	-2.56743800	1.06829200
H	3.81582900	-2.48230800	1.64095800
H	2.43435400	-3.54031100	1.27942700
C	-2.45538400	3.25760900	0.99467600
H	-1.98119400	3.78136400	1.83019600
H	-2.94655200	4.00424900	0.36232600
C	-3.43429900	2.22260700	1.52899100
H	-4.34852700	2.69144900	1.92807200
H	-2.96038000	1.63385600	2.31941000
N	-3.81123900	1.24627600	0.44427800
C	-4.59996700	1.92175200	-0.64630000
H	-5.40967600	2.50884200	-0.18512200
H	-3.91597600	2.60258800	-1.16067200
C	-5.20326200	0.90398500	-1.62221500
H	-5.59856000	1.45141700	-2.48603700
H	-6.06686500	0.41320000	-1.15747100
C	-4.22745500	-0.15911200	-2.14203300
H	-4.79517300	-0.92000000	-2.69999100
H	-3.48562300	0.28280400	-2.81455200
C	-4.57341400	0.07856400	1.01243800
H	-5.63319800	0.34521800	1.12652700

H	-4.15848400	-0.12071300	2.00237700
C	-4.37292000	-1.17596400	0.12072900
H	-3.86814500	-1.94849400	0.70356000
H	-5.32575900	-1.58422200	-0.24497000
N	-3.46735300	-0.84638200	-1.03241900
C	-2.77216000	-2.07516600	-1.55530700
H	-3.46079400	-2.64486100	-2.19764100
H	-2.50573700	-2.68119500	-0.68506900
C	-1.49731800	-1.68808200	-2.28668000
H	-1.68918600	-1.14912100	-3.22021500
H	-0.90473800	-2.58066800	-2.51131300
H	0.54762800	2.09150500	1.18382400

[Ni$_2$-Fe-SHS-2]$^+$

1 1

Ni	-2.65626300	-0.19416000	-0.38113000
Ni	2.16761800	0.70374200	-0.21175300
Fe	0.41960200	-2.08640300	0.35658700
S	-2.39431800	-0.34824700	-2.57404500
S	-1.83585400	-2.18068700	0.05601000
S	0.96019200	-0.65664100	-1.41249900
S	0.55791200	2.13792600	0.13558100
O	1.48205400	-4.63862200	-0.23838100
O	1.26702700	-1.21859600	2.92031800
N	-3.40020200	1.64784600	-0.57316900
N	-3.22757200	0.06015700	1.47253700
N	0.98462500	-3.57695700	-0.06052800
N	0.84244100	-1.47459000	1.83167700
C	-2.99335000	1.35610700	-3.01911500
H	-2.40793100	1.72362300	-3.86583200
H	-4.02525100	1.21637700	-3.35358300
C	-2.87623300	2.29367600	-1.82078600
H	-1.82279600	2.52453500	-1.63154200
H	-3.41868400	3.23248100	-2.01716800
C	-4.89379700	1.46590300	-0.60981900
H	-5.11811000	0.70073200	-1.36045500
H	-5.36362100	2.41167000	-0.92437200
C	-5.44822300	1.05843100	0.76369200
H	-6.50051100	0.77831600	0.63576500
H	-5.44852700	1.92802200	1.43161400
C	-4.72795300	-0.11341800	1.44665100
H	-5.08867700	-0.19763600	2.48370100
H	-4.93584000	-1.05624700	0.93227700
C	-2.99550300	2.42202200	0.64805300
H	-3.71338200	3.23211900	0.83602800
H	-2.01611200	2.85786900	0.43678800
C	-2.85037200	1.46346200	1.85818300
H	-1.79638200	1.43142100	2.14564700
H	-3.44691200	1.78904500	2.72232100

C	-2.58288700	-0.93962400	2.39979400
H	-3.10862100	-0.92904700	3.36748700
H	-1.55086300	-0.61094400	2.54609600
C	-2.58085000	-2.31301900	1.75888700
H	-3.58549200	-2.73568900	1.65166000
H	-1.96877900	-3.01185900	2.33475000
C	2.39287000	-1.48709900	-2.26800500
H	2.05733600	-2.45958000	-2.63994900
H	2.68327500	-0.86249700	-3.11889600
C	3.50839500	-1.65755900	-1.24995700
H	4.42904200	-2.05145600	-1.70613300
H	3.16915300	-2.34444000	-0.47118000
N	3.80209000	-0.34109900	-0.57702600
C	4.66103000	0.51576900	-1.47496600
H	5.48245500	-0.10372100	-1.86713100
H	4.03587000	0.83288300	-2.31556600
C	5.25445800	1.72358600	-0.74219000
H	5.73182500	2.37282300	-1.48570000
H	6.05833100	1.39050000	-0.07429700
C	4.24233000	2.56227200	0.04409900
H	4.78151200	3.29227200	0.66782000
H	3.57215600	3.10311900	-0.62991500
C	4.45511900	-0.54300400	0.76164200
H	5.52770900	-0.74976900	0.63832200
H	3.98531600	-1.42123000	1.20888800
C	4.19608700	0.68806500	1.67298500
H	3.60854700	0.37274300	2.53783100
H	5.13005300	1.14051800	2.03501300
N	3.37526200	1.71374100	0.94006900
C	2.60559400	2.58331300	1.90258900
H	3.31020000	3.24855700	2.42809300
H	2.13720500	1.90863700	2.62487600
C	1.52956300	3.35112600	1.15285100
H	1.95143800	4.13094100	0.50962800
H	0.85708600	3.83569100	1.86868900
H	-1.03338200	-0.10621200	-2.57078800

SI References:

1. J. J. Smee, M. L. Miller, C. A. Grapperhaus, J. H. Reibenspies and M. Y. Darensbourg, *Inorg. Chem.*, 2001, **40**, 3601-3605.
2. D. K. Mills, J. H. Reibenspies and M. Y. Darensbourg, *Inorg. Chem.*, 1990, **29**, 4364-4366.
3. L. Hedberg, K. Hedberg, S. K. Satija and B. I. Swanson, *Inorg. Chem*., 1985, **24**, 2766.
4. D. W. McBride, S. L. Stafford and F. G. A. Stone, *Inorg. Chem.*, 1962, **1**, 386–388.
5. *APEX2*, Bruker AXS Inc., Madison, WI, 2007.
6. *SAINT*, Bruker AXS Inc., Madison, WI, 2007.
7. G. Sheldrick, *Acta Crystallographica Section A*, 2008, **64**, 112.
8. L. J. Barbour, *J. Supramol. Chem.*, 2003, **1**, 189.
9. G. A. Bain and J. F. Berry, *J. Chem. Educ*., 2008, **85**, 532-536.
10. A. M. Appel and M. L. Helm, *ACS Catal*., 2014, **4**, 630.
11. S. Wiese, U. J. Kilgore, M. –H. Ho, S. Raugei, D. L. DuBois, R. M. Bullock and M. L. Helm, *ACS Catal*., 2013, **3**, 2527
12. V. Fourmond, P. A. Jacques, M. Fontecave and V. Artero, *Inorg. Chem*., 2010, **49**, 10338.
13. S. Daniele, P. Ugo, G. –A. Mazzocchin and G. Bontempelli, *Anal. Chim. Acta*,1985, **173**,141
14. G. A. N. Felton, R. S. Glass, D. L. Lichtenberger and D. H. Evans, *Inorg. Chem*., 2006, **45**, 9181
15. S. Ding, P. Ghosh, A. M. Lunsford, N. Wang, N. Bhuvanesh, M. B. Hall and M. Y. Darensbourg, *J. Am. Chem. Soc.*, 2016, **138**, 12920.
16. P. Ghosh, M. Quiroz, N. Wang, N. Bhuvanesh and M. Y. Darensbourg, *Dalton Trans.*, 2017, **46**, 5617.
17. C. –H. Hsieh, S. Ding, O. F. Erdem, D. J. Crouthers, T. Liu, C. C. L. McCrory, W. Lubitz, C. V. Popescu, J. H. Reibenspies, M. B. Hall and M. Y. Darensbourg, *Nat. Commun*., 2014, **5**, 3684.
18. Tao, J.; Perdew, J. P.; Staroverov, V. N.; Scuseria, G. E. *Phys. Rev. Lett.*, **2003**, *91*, 146401.
19. M. J. Frisch, G. W. Trucks, H. B. Schlegel, G. E. Scuseria, M. A. Robb, J. R. Cheeseman, G. Scalmani, V. Barone, B. Mennucci, G. A. Petersson, H. Nakatsuji, M. Caricato, X. Li, H. P. Hratchian, A. F. Izmaylov, J. Bloino, G. Zheng, J. L. Sonnenberg, M. Hada, M. Ehara, K. Toyota, R. Fukuda, J. Hasegawa, M. Ishida, T. Nakajima, Y. Honda, O.Kitao, H. Nakai, T. Vreven,J. A. Montgomery Jr., J. E. Peralta, F. Ogliaro, M. J. Bearpark, J. Heyd, E. N. Brothers, K. N. Kudin, V. N. Staroverov, R. Kobayashi, J. Normand, K. Raghavachari, A. P. Rendell, J. C. Burant, S. S. Iyengar, J.Tomasi, M. Cossi, N. Rega, N. J. Millam, M. Klene, J. E. Knox, J. B. Cross, V. Bakken, C. Adamo, J. Jaramillo, R. Gomperts, R. E. Stratmann, O. Yazyev, A. J. Austin, R. Cammi, C. Pomelli, J. W. Ochterski, R. L. Martin, K. Morokuma, V. G.Zakrzewski, G. A. Voth, P. Salvador, J. J. Dannenberg, S. Dapprich, A. D. Daniels, Ö. Farkas, J. B. Foresman, J.V. Ortiz, J. Cioslowski, D. J. Fox, *Gaussian 09*, Gaussian, Inc.: Wallingford, CT, USA, 2009.
20. S. M. Brothers, M. Y. Darensbourg and M. B. Hall, *Inorg. Chem*., 2011, **50**, 8532-8540.
21. R. Krishnan, J. S. Binkley, R. Seeger and J. A. Pople, *J. Chem. Phys*.,1980,**72**, 650-654.
22. A. D. McLean and G. S. Chandler, *J. Chem. Phys*., 1980, **72**, 5639-5648.
23. T. Clark, J. Chandrasekhar, G. W. Spitznagel and P. V. R. Schleyer, *J. Comput. Chem*., 1983, **4**, 294-301.
24. A. J. H. Wachters, *J. Chem. Phys*., 1970, **52**, 1033-1036.
25. P. J. Hay, *J. Chem. Phys*., 1977,**66**, 4377-4384.
26. K. Raghavachari and G. W. Trucks, *J. Chem. Phys*., 1989, **91**, 1062-1065.
27. A. V. Marenich, C. J. Cramer and D. G. Truhlar, *J. Phys. Chem. B*, 2009, **113**, 6378-6396.

28. J. P. Foster andF. Weinhold, *J. Am. Chem. Soc.*, 1980, **102**, 7211-7218.
